# The population structure of vancomycin-resistant and -susceptible *Enterococcus faecium* in a low-prevalence antimicrobial resistance setting is highly influenced by circulating global hospital-associated clones

**DOI:** 10.1099/mgen.0.001160

**Published:** 2023-12-19

**Authors:** Mushtaq AL Rubaye, Jessin Janice, Jørgen Vildershøj Bjørnholt, Oliver Kacelnik, Bjørg C. Haldorsen, Randi M. Nygaard, Joachim Hegstad, Arnfinn Sundsfjord, Kristin Hegstad, the Norwegian VRE study group

**Affiliations:** ^1^​ Research group for Host–Microbe Interactions, Department of Medical Biology, UiT The Arctic University of Norway, Tromsø, Norway; ^2^​ Norwegian National Advisory Unit on Detection of Antimicrobial Resistance, Department of Microbiology and Infection Control, University Hospital of North Norway, Tromsø, Norway; ^3^​ Department of Clinical Microbiology, Oslo University Hospital, Oslo, Norway; ^4^​ Institute of Clinical Medicine, University of Oslo, Oslo, Norway; ^5^​ Department of Antibiotic Resistance and Infection Prevention, Norwegian Institute of Public Health, Oslo, Norway; ^6^​ Department of Microbiology, Haukeland University Hospital, Bergen, Norway; ^7^​ Department of Microbiology and Infection Control, University Hospital of North Norway, Tromsø, Norway; ^†^​ Present address: Section for development, Department of Microbiology, Clinic for Laboratory Medicine, Oslo University Hospital, Oslo, Norway

**Keywords:** *E. faecalis*, *E. faecium*, *vanA* gene cluster, *vanB* gene cluster, vancomycin-resistant enterococci, VRE outbreak

## Abstract

Between 2010 and 2015 the incidence of vancomycin-resistant *Enterococcus faecium* (VRE*fm*) in Norway increased dramatically. Hence, we selected (1) a random subset of vancomycin-resistant enterococci (VRE) from the Norwegian Surveillance System for Communicable Diseases (2010–15; *n*=239) and (2) Norwegian vancomycin-susceptible *E. faecium* (VSE*fm*) bacteraemia isolates from the national surveillance system for antimicrobial resistance in microbes (2008 and 2014; *n*=261) for further analysis. Whole-genome sequences were collected for population structure, *van* gene cluster, mobile genetic element and virulome analysis, as well as antimicrobial susceptibility testing. Comparative genomic and phylogeographical analyses were performed with complete genomes of global *E. faecium* strains from the National Center for Biotechnology Information (NCBI) (1946–2022; *n*=272). All Norwegian VRE*fm* and most of the VSE*fm* clustered with global hospital-associated sequence types (STs) in the phylogenetic subclade A1. The *vanB2* subtype carried by chromosomal Tn*1549* integrative conjugative elements was the dominant *van* type. The major Norwegian VRE*fm* cluster types (CTs) were in accordance with concurrent European CTs. The dominant *vanB*-type VRE*fm* CTs, ST192-CT3/26 and ST117-CT24, were mostly linked to a single hospital in Norway where the clones spread after independent chromosomal acquisition of Tn*1549*. The less prevalent *vanA* VRE were associated with more diverse CTs and *vanA* carrying Inc18 or RepA_N plasmids with toxin–antitoxin systems. Only 5 % of the Norwegian VRE were *Enterococcus faecalis,* all of which contained *vanB*. The Norwegian VRE*fm* and VSE*fm* isolates harboured CT-specific virulence factor (VF) profiles supporting biofilm formation and colonization. The dominant VRE*fm* CTs in general hosted more virulence determinants than VSE*fm*. The phylogenetic clade B VSE*fm* isolates (*n*=21)*,* recently classified as *Enterococcus lactis *, harboured fewer VFs than *E. faecium* in general, and particularly subclade A1 isolates. In conclusion, the population structure of Norwegian *E. faecium* isolates mirrors the globally prevalent clones and particularly concurrent European VRE*fm*/VSE*fm* CTs. Novel chromosomal acquisition of *vanB2* on Tn*1549* from the gut microbiota, however, formed a single major hospital VRE*fm* outbreak. Dominant VRE*fm* CTs contained more VFs than VSE*fm*.

## Data Summary

Illumina and PacBio reads and/or assemblies are available under the following project numbers: PRJNA858233, PRJNA407052, PRJNA393251 and PRJNA306646. Biosample ID and metadata are provided in File S1, available in the online version of this article. The authors confirm all supporting data, code and protocols are provided within the article or through supplementary data files.

**Impact Statement**

This study represents the first comprehensive unveiling of the population structure of *Enterococcus faecium* in a low-prevalence antimicrobial resistance setting, including both vancomycin-resistant (VRE*fm*) and -sensitive (VSE*fm*) isolates. Through comparative genomic analysis we have provided new insights into the epidemiology and population structure of and interaction between VRE*fm* and VSE*fm*, highlighting critical factors for the understanding and prevention of VRE spread. Importantly, our study discloses the virulome profiles of VRE*fm* and VSE*fm* using an in-house database of 30 experimentally verified virulence factors involved in *E. faecium* pathogenesis. VRE*fm* exhibited higher virulence factor content than genetically related VSE*fm*. The overall findings expand our current knowledge of the epidemiology and spread of VRE*fm* and provides new insights into the genomic evolution of clinical strains of VRE*fm* and VSE*fm*. Finally, we demonstrated the minor role played by *Enterococcus faecalis* in the spread of VRE in a low-AMR-prevalence setting.

## Introduction

*Enterococcus faecium* and *Enterococcus faecalis* are opportunistic pathogens residing in the human gut microbiota. They can cause severe infections in immunocompromised hospitalized patients [1]. The remarkable adaptability of enterococcal genomes and their capacity to acquire antimicrobial resistance (AMR) genes have played a pivotal role in transforming them into increasingly important opportunistic pathogens [2–4]. Although *E. faecalis* causes most infections, the hospital-adapted *E. faecium* genotype is more prone to develop multidrug resistance (MDR) [3]. The global phylogeny of *E. faecium* is characterized by the dominance of two distinct phylogenetic clades, A and B. Clade A can be further divided into two subclades: A1 consisting primarily of clinical strains, and A2 consisting of strains mainly found in animals but also some non-hospitalized individuals. Clade B encompasses community isolates [4–6] and was recently reclassified as *Enterococcus lactis* [7].

*E. faecium* infections are difficult to treat because of both intrinsic and acquired antimicrobial resistance. Vancomycin is a preferred drug in treating *E. faecium* infections [1]. The increasing prevalence of enterococcal infections has been associated with a rise of vancomycin resistance [8]. Ten different *van* gene clusters (*vanA*, *vanB*, *vanC*, *vanD*, *vanE*, *vanG*, *vanL*, *vanM*, *vanN* and *vanP*) are responsible for vancomycin resistance in enterococci [9]. The *vanC* gene cluster is intrinsic in *Enterococcus casseliflavus* and *Enterococcus gallinarum* [3, 10], while the other *van* gene clusters have been associated with acquired vancomycin resistance only [9, 11].

*VanA-* and *vanB-*type vancomycin-resistant enterococci (VRE) are the most prevalent worldwide and are predominantly found in vancomycin-resistant *E. faecium* (VRE*fm*) [3]. While the *vanA* gene cluster is usually part of the Tn*1546* transposon and often found on plasmids [12], the widespread *vanB2* subtype gene cluster is associated with Tn*1549* integrative conjugative elements (ICEs) originally acquired from gut anaerobes [13]. However, the mechanisms driving the dissemination of VRE*fm* are complex and both clonal spread and exchange of mobile genetic elements (MGEs) likely play important roles [14].

Although *E. faecium* and *E. faecalis* are not considered highly virulent, both species possess virulence factors (VFs) associated with colonization, host invasion and/or tissue damage [3, 15], or otherwise bypassing the host immune system [16]. In *E. faecium* most of the VFs are involved in interactions with the extracellular matrix proteins vital in biofilm formation and colonization [17].

Since 1996, clinical infections and carriage of VRE have been notifiable to the Norwegian Surveillance System for Communicable Diseases (MSIS). VRE is defined as *E. faecium* or *E. faecalis* harbouring *van* gene clusters. The annual number of reported VRE cases was <10 before 2010. After 2010, there was a significant increase in the prevalence of VRE, reaching a peak in 2011, followed by a subsequent decrease, although it never returned to the pre-2010 levels. In addition, the Norwegian surveillance system for antibiotic resistance in microbes (NORM/NORM-VET) systematically collects and monitors antimicrobial susceptibility data in human and animal pathogens, including *E. faecium* and *E. faecalis* [18]. Vancomycin-susceptible *E. faecium* and *E. faecalis* are defined according to European Committee on Antimicrobial Susceptibility Testing (EUCAST) breakpoints and for *E. faecium* additionally absence of *vanA* and *vanB*. The nationwide programmes ensure standardized collection, antimicrobial susceptibility testing and storage of strains, providing a unique opportunity to obtain both vancomycin-susceptible and -resistant enterococcal isolates for further investigation.

In this study, we aimed (i) to perform a comparative phylogenomic analysis of the Norwegian VRE from 2010–15, invasive Norwegian vancomycin-susceptible *E. faecium* (VSE*fm*; 2008 and 2014) and global strain genomes, and (ii) describe the dominant VRE outbreak clones, their MGEs harbouring *van* gene clusters and the VF profile of *E. faecium *.

## Methods

### Samples size, collection descriptions and data collection

A total of 502 *E. faecium* (*n*=469), *E. faecalis* (*n*=12) and *E. lactis* (*n*=21) isolates from two different collections were included. (1) Randomly selected clinical and screening isolates of VRE (2010–throughout June 2015) from MSIS [19–21]. The study period was chosen because of a sudden increase in VRE incidence from 2010 (0.12 in 2009, 1.10 in 2010 and 5.87 cases in 2011 per 100 000 person years), which then gradually decreased to 1.5 in 2015. (2) Blood culture isolates of vancomycin-susceptible enterococci (VSE) from [19] and [20] and inclusion of the VSE collection allow us to compare vancomycin-susceptible *E. faecium* (VSE*fm*) and VRE*fm* genomes, before and after the increase of VRE. Ninety per cent of the VSE isolates from [19] and 93 % of the VSE isolates from [20] were available for inclusion (Table 1). The VSE collection included 21 *E. lactis* isolates previously identified as *E. faecium *. The VRE collection consisted of 239 isolates of the 783 (31 %) VRE reported to MSIS between 2010 and 2015, of which 87 (11 %) were from clinical infections. The relative proportion of included VRE compared to the total numbers of VRE reported in Norway is illustrated in Fig. S1. The VRE collection included all of the clinical isolates. Weighted across geography and time, up to three faecal carrier isolates per clinical isolate were selected. If there was no clinical isolate in the geography and time category, a random carrier isolate was selected as index [22]. Twenty-two isolates were excluded from this study (5 due to wrong species identity, 14 because they were not available for sequencing and 3 because of repeated low quality of their assemblies). Thus, a total of 227 VRE*fm* and 12 VRE*fs* were included in the study. In addition, two VRE*fm* isolates recovered in 1996 from the first VRE outbreak reported in Norway [23] were included in the phylogenetic analyses (Table 1). All of the isolates in the study are listed in File S1 with anonymized IDs, and the names of hospitals have been changed to IDs comprising a letter (N, C, E and W, referring to the Northern, Central, South-Eastern and Western health regions of Norway, respectively) and a digit. An overview of sequence types (STs) for VRE from 2019 to 20 was obtained from the Norwegian National Advisory Unit on Detection of Antimicrobial Resistance (K-res) to compare the 2010–15 ST distribution to more recent data.

**Table 1.** Bacterial isolates included in the study and proportions of phenotypic resistance to ampicillin, linezolid and high-level gentamicin resistance (HLGR)

Collection and year	Isolates, *n*	Ampicillin resistant, *n* (%)	HLGR, *n* (%)	Linezolid resistant, *n* (%)
**VRE**				
* E. faecium* 2010–2015	227	226 (99.5)	82 (36)	1 (0.4)
* E. faecalis* 2010–2015	12	0	8 (67)	0
* E. faecium* 1996	2	2 (100)	0	0
**VSE**				
* E. faecium* 2008	93	82 (88)	55 (59)	0
* E. lactis* 2008	6	1 (17)	0	0
* E. faecium* 2014	147	138 (94)	61 (41)	0
* E. lactis* 2014	15	1 (7)	0	1 (7)

### Species identification and antimicrobial susceptibility testing (AST)

A single blood agar culture colony was used for sub-culturing and subsequent AST, genomic DNA extraction for whole-genome sequencing and species identification by matrix-assisted laser desorption/ionization time-of-flight mass spectometry (MALDI-TOF MS) (Bruker Daltonics GmbH, Bremen, Germany). For the VSE, AST data were collected as part of the NORM programme (appendix 5 in the NORM report) [20]. For the VRE, AST was performed at K-res using the same methods as in NORM, performed and interpreted according to the EUCAST disc diffusion method [24], and EUCAST clinical breakpoints [25], respectively. The Clinical and Laboratory Standards Institute (CLSI) agar screening method was used for detection of reduced susceptibility to vancomycin [26].

### Whole-genome sequencing

Initially, all samples were subjected to short-read sequencing. First, the DNeasy Blood and tissue kit (Qiagen, Hilden, Germany) was used to extract the genomic DNA. Next, a Qubit fluorometer (Invitrogen) was used to quantify the concentration of total genomic DNA. The Genomics Support Center Tromsø sequenced the samples using the Illumina NextSeq550 system as described previously [27]. A selection of 21 isolates was subsequently chosen for long-read sequencing to use as reference genomes. The selection was based on their position in the phylogenetic tree. The Wizard Genomic DNA Purification kit (Promega, Madison, USA) was used to extract a large quantity of genomic DNA for long-read sequencing. Then, the genomic DNA concentration was quantified with a Qubit fluorometer. Long-read sequencing was performed at the Norwegian Sequencing Centre (University of Oslo). To prepare multiplexed microbial libraries, the SMRTbell Express Template prep kit 2.0 was used according to the Pacific Biosciences (PacBio) protocol. Fragmentation of DNA was carried out using g-tubes (Covaries) resulting in 10–16 kb-sized fragments. To select the final library, BluePippin with an 8 kb cut-off was used. Libraries were sequenced on ~90 % of the 8M SMRT cell on Sequel II using the Sequel II Banding kit 2.0 and sequencing chemistry v2.0. Demultiplex barcodes pipeline was carried out using SMRT Tools (SMRT Link v9.0.0.92188) to demultiplex the reads (minimum barcode score 26). Finally, the circular consensus sequencing (CCS) sequences were produced for demultiplexed data using CCS pipeline (SMRT Link v9.0.0.92188). The resulting PacBio reads length ranged from 10 to 20 kb.

### Genomic analyses

For Illumina-sequenced samples Trimmomatic v0.39 was used to perform quality trimming and adaptor removal [28] before output reads files were assessed using FastQC [29].

Next, Unicycler v0.4.7 was used for genome assembly [30] and, finally, quality assessment of the genome assemblies was performed using Quast v5.0.2 [31]. A cut-off maximum of 400 contigs and a minimum of 40× genome coverage were used for Illumina-sequenced samples to consider the assemblies as eligible to be included in the analyses (with the exception of three samples with 30–37× coverage). Moreover, the genome size should not show more than ±10 % fluctuation compared to the smallest and largest complete *E. faecium* or *E. faecalis* genome assemblies in the National Center for Biotechnology Information’s (NCBI’s) Refseq database.

For PacBio-sequenced samples, Unicycler was used to assemble the CCS reads. The assemblies that Unicycler was unable to circularize were reassembled using Canu v2.2 [32], corrected with Pilon v1.23 [33] and circularized using circulator v1.5.5 [34]. Finally, we performed quality assessment using QUAST. The prokaryotic genome annotation pipeline of NCBI was used to annotate the assemblies, MGEs and plasmids [35]. Snippy v3.1 was used for variant calling between sequences [36].

### Multilocus sequence typing (MLST)

MLST was carried out for all samples using MLST v2.19.0 [37]. To generate minimum spanning trees, core genome MLST was performed using SeqSphere+ software v6.0.2 (Ridom GmbH, Münster, Germany; http://www.ridom.de/seqsphere/). For *E. faecium* isolates, the scheme included 1423 core genes and a threshold of ≤20 allelic differences for cluster calculation and determination of clonal relatedness [38]. The scheme of 1972 gene targets with ≤7 allelic differences was set up for cluster calculation and clonal relatedness of *E. faecalis* genomes [39]. Novel STs and cluster types (CTs) were obtained by submission of assemblies for allelic profiling to PubMLST [40] and Ridom SeqSphere+, respectively.

### Phylogenetic trees

Phylogenetic trees based on the core genome of the Norwegian *E. faecium* and *E. lactis* were constructed using Parsnp v1.2 [41]. The global tree included all Norwegian *E. faecium* and *E. lactis* isolates of the study (*n*=490), as well as all publicly available complete genomes of *E. faecium* retrieved from the NCBI as of 11 May 2022 (*n*=272). In addition, a local tree that only included the 490 Norwegian *E. faecium* and *E. lactis* was built. Finally, Interactive Tree Of Life (iTOL) was applied to display metadata in the trees [42].

### MGEs harbouring the *vanB* gene cluster

To identify the *van* type in the VRE assemblies, the NCBI bacterial AMR reference gene database (PRJNA313047) was used in the ABRicate tool v1.0.1 [43]. To locate and extract the sequences of MGEs harbouring *vanB* gene clusters in individual isolates, the closest PacBio closed VSE genome was used as a reference. The contigs of the Illumina assemblies were sorted according to the references using Mauve [44]. Next, sorted Illumina assemblies were concatenated and blasted against their reference genomes using the basic local alignment search tool (blastn) v2.6.0 [45]. The Artemis Comparison Tool (ACT) [46] was used to visualize the blasts and locate the MGEs harbouring the *vanB* gene cluster. Finally, one representative from each MGE type was chosen to perform a blast and visualize the results using Easyfig v2.2.2 [47].

### Plasmids harbouring the *vanA* gene cluster

Mob-suite was used to reconstruct plasmids in VanA-type VRE*fm* isolates [48]. Plasmid typing was performed using the PlasmidFinder v2.0.1 online database (https://cge.food.dtu.dk/services/PlasmidFinder/). Then plasmids were blasted against the NCBI bacterial AMR reference gene database (PRJNA313047) using the ABRicate tool v1.0 to find those containing the *vanA* gene cluster. To compare the plasmids and determine the identity between them, a closed PacBio-sequenced *vanA* plasmid of each cluster type was utilized as a reference for reads mapping. The mem algorithm in the BWA tool v07.17 [49] was used to map the reads against the reference sequence. Indexing and sorting were performed in SAMtools v1.10 [50] and the resulting BAM file was visualized using Artemis v18.1.0 [46]. Samples whose reads fully covered the reference *vanA* plasmid were considered to contain plasmids similar to the reference. EasyFig v2.2.2 was used to blast the closed plasmids and generate a comparison figure.

### Virulence factor profile

All of the *E. faecium* and *E. lactis* genomes were investigated for the presence of the determinants of 30 experimentally confirmed VFs (File S2) [17, 51–60]. The coding sequences of all 30 VFs were used to build a database in ABRicate v1.0.1 [43]. blasting of the *E. faecium* and *E. lactis* genomes against the database was performed using the minimum cut-off for identity and coverage at 90 %. Next, the local phylogenetic tree of *E. faecium* was annotated using iTOL [42]. Since the *esp* gene contains several repeats [61], only the conserved part of this gene (2190 bp) was used to blast against the assemblies. For *scm*, a new allele was found in our samples; the new allele is 173 bp longer than the reference allele. These extra nucleotides are in the linker region and between the two conserved domains of the gene. For *scm*, both alleles were used for blast searches.

## Results and discussion

### Both Norwegian VRE*fm* and VSE*fm* are dominated by prevalent global STs

**Fig. 1.**
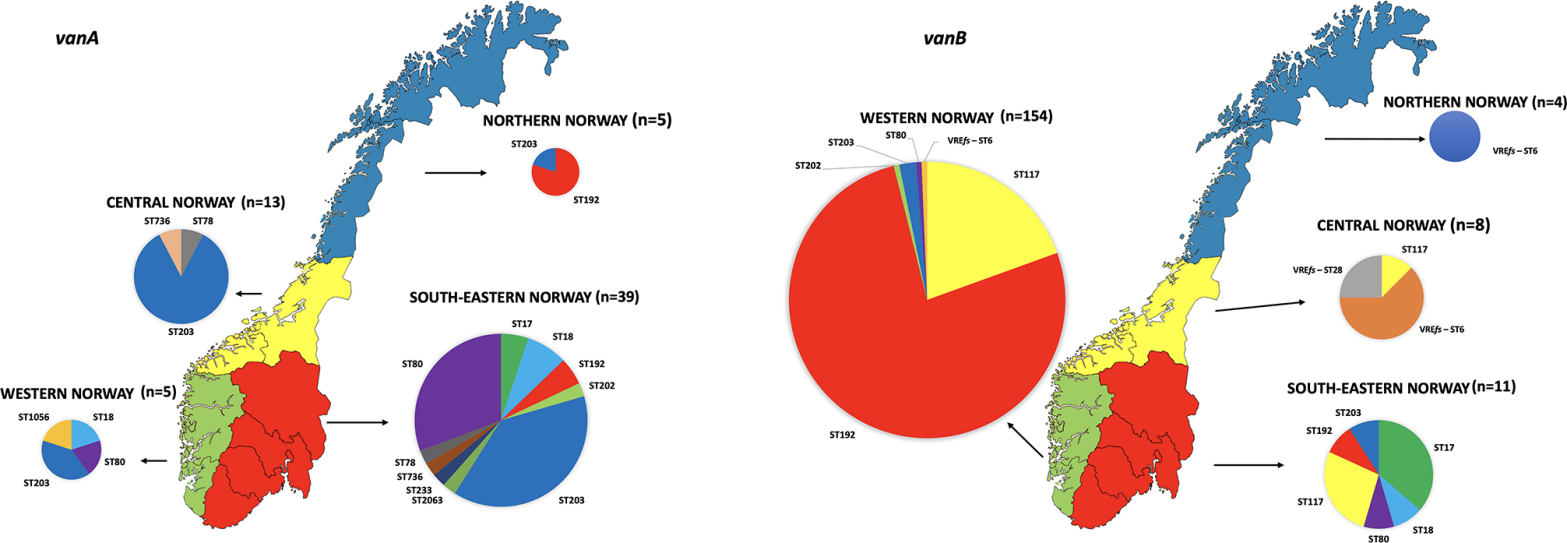
Number of VRE of different STs by health region in the VRE 2010–2015 collection. The map at the left shows the *vanA* and the one at the right shows the *vanB* ST distribution. The four health regions of Norway are coloured in the maps, and pie charts illustrate the frequency of different STs in each region. The STs of the VRE *E. faecalis* (VRE*fs*) *vanB* are specified.

**Fig. 2.**
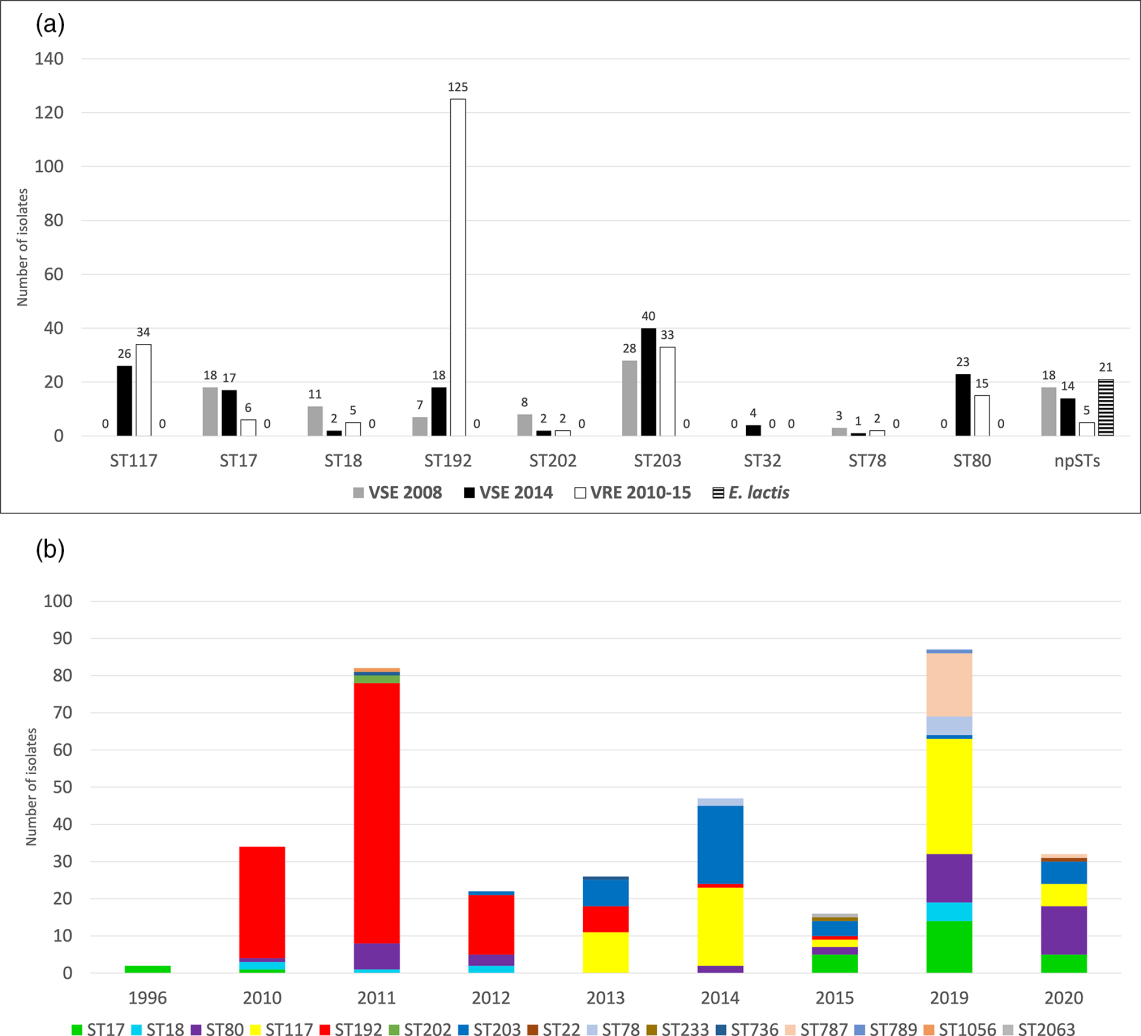
The frequencies of STs based on collection and year. (a) Frequencies of *E. faecium* STs shown per sample collection (VSE 2008, VSE 2014 and VRE2010–15) and of VSE *E. lactis *. The chart illustrates the STs containing at least 1 % of the total number of isolates in this study. STs with <1 % are shown together as non-prevalent STs (npSTs). (b) The prevalence of STs per year shown for VRE*fm*. Data for 2019 and 2020 were added to compare shifts of STs from the period of the study (2010–2015) to more recent data (2019–2020).

Out of the VRE*fm* 2010–15 isolates, 165 were identified as *vanB* type, while 62 were identified as *vanA* type (Fig. 1 and File S1). The majority of the VRE*fm* 2010–15 isolates (*n*=227) were classified as ST192 (55 %), followed by ST117 (15 %), ST203 (14 %), ST80 (7 %) and ST17 (3 %). Non-prevalent STs (npSTs), including ST18, ST78 and ST202, amounted to 6 % (Figs 1 and 2a). A marked shift in the relative proportions of STs was observed when comparing the VRE*fm* 2010–15 isolates to Norwegian VRE data from 2019 to 2020 [18, 62] (Fig. 2b). The incidence of VRE in 2019 and 2020 was 3.82 and 1.39 cases per 100 000 person years, respectively. While VRE*fm* ST192 was most dominant during 2010–12, it was not observed in 2019–20. In contrast, the prevalence of VRE*fm* ST17 and ST80 increased in the latter years and ST117 started to appear in 2013. All the prevalent STs have been or still are among the dominant STs in European countries. For instance, ST192 was a globally dominant ST mostly related to *vanB* type VRE in the 2010s [63–65]. ST117 was a dominant ST in Germany over the 1990s and its prevalence increased again after 2010 [63, 66]. ST80 was responsible for the largest VRE outbreak recorded in Germany between 2015 and 2017 with 2900 (*vanB*-type) cases. ST203, ST17, and ST18 were among the most common STs in Germany from 2000 to 2009, but they began to fade away after a decade (2010–19) [63]. Overall, the major VRE*fm* STs from 2010 were gradually replaced by other STs, showing clonal sweeps of new STs and ST reintroduction (Fig. 2b), consistent with observations from other countries, including Germany and Denmark [63, 67].

The main VSE*fm* STs are the same as in VRE*fm* 2010–15 but in a different order of prevalence; ST203 (26 %), ST17 (13 %), ST117 (10 %), ST192 (10 %), ST80 (9 %) and ST18 (5 %). The npSTs, including ST32, ST78 and ST202, as well as the 21 *E. lactis* isolates, covered 27 % of the VSE (Fig. 2a). The presence of each ST varied over time and between VRE*fm* and VSE*fm*. For instance, ST80 and ST117 were absent among VSE*fm* 2008 but appeared in VRE*fm* in 2010 and 2013, respectively, and became prevalent STs in VSE*fm* 2014. ST203, another dominant VRE*fm* ST in 2014, was also present in VSE*fm* 2014. In contrast, ST17, ST18, ST32 and ST202 were present in VSE*fm* 2014 but absent in VRE of the same year. Moreover, in VRE 2014, only two isolates out of 47 belonged to the npSTs, while in VSE*fm* 2014, 34 out of 162 isolates were npSTs, including 15 *E. lactis* (Fig. 2). Thus, the VSE*fm* are much more diverse in STs, while the VRE*fm* primarily belong to typical global STs.

### Norwegian VRE*fm* are dominated by concurrent major European clusters

In total, 25 *vanA*-type CTs (19 singletons) and 19 *vanB*-type CTs (12 singletons) were detected (Fig. S2; Files S1 and S3). The higher diversity and wider geographical dispersion of *vanA*-type CTs were consistent with smaller outbreaks. We identified four major Norwegian hospital VRE outbreaks during the study period: the *vanB*-type ST192-CT3/CT26 and ST117-CT24 (Table 2 and File S3), and the *vanA*-type ST203-CT20 and ST80-CT3097 (Table 3 and File S3). The most prevalent VSE*fm* CTs are the mixed *vanB* VRE–VSE clusters, ST117-CT24, ST203-CT3061 and ST80-CT16 (File S3). Three of the predominant VRE*fm* clusters (the *vanB*-type ST192-CT3/CT26 and ST117-CT24 and *vanA*-type ST203-CT20) have been reported in other European countries (see Document S1 for details).

**Table 2.** Characteristics of Norwegian VRE*fm* clusters and their *vanB* gene-harbouring MGEs

Cluster	Isolates*, *n*	MGE	MGE insertion location	Insertion sequence on reference genome (5’–3’)
*** E. faecium ***				
ST192-CT3/CT26	113	Tn*1549*	*sir* gene of *tirE* operon	AATATTAAAGGAA
ST117-CT24	31	Tn*1549*	*btuD* gene encoding vitamin B12 import ATP-binding protein	AAAAGTTTTT
ST203-CT3061	3	Tn*1549*	Between two CDSs encoding hypothetical proteins (HPs)	TTTTTATAAAAAAA
ST17-CT1709	2	Tn*1549*	Between CDSs encoding ribonucleoside-diphosphate reductase 2 subunit beta and HP	TTCAAAAATTTT
ST17-CT6207	1	Tn*1549*	IS*3* family transposase gene	TTTTTTCTTAAAA
ST80-CT16	1	Tn*1549*	Between tRNA-Gly and CDS encoding HP	ATTTTACT
*** E. faecalis ***				
ST6-CT107	4	Plasmid	CDS encoding HP	GATGATGT
ST6-CT1160	3	Tn*1549*	Between peptidase propeptide and oligopeptide-binding protein (*oppA*) genes	TTTTGACA
ST28-CT1162	2	Tn*1549*	CDS encoding catechol-2,3-dioxygenase	TTTTAT

*Singleton VRE*fs* isolates and 15 VRE*fm* isolates with low-quality assembly in the insertion site of Tn*1549* are not included in this table.

**Table 3.** Characteristics of *vanA* gene clusters and plasmids in the PacBio-sequenced Norwegian VRE*fm*

CT (Reference isolate)	Isolates, *n*	Plasmid size	CDSs, *n*	Plasmid type	Toxin–antitoxin systems	Transposon in plasmid
ST203-CT20 (51271218)	19	55 kb	73	Inc18	Epsilon–Zeta	Tn*552*
ST80-CT3097 (51271936)	10	32 kb	42	RepA_N (rep17)	Axe–Txe	Tn*1546*
ST192-CT188 (51271057)	4	62 kb	72	Inc18	Epsilon–Zeta	Tn*1546*
ST18-CT3042 (51276509)	2	43 kb	51	RepA_N (rep17)	Axe–Txe	
ST17-CT3037 (51271928)	2	38 kb	47	RepA_N (rep17)	Axe–Txe and Epsilon–Zeta	
ST202-CT3079 (51271933)	1	35 kb	43	RepA_N (rep17)	Axe–Txe	Tn*1546*

### *vanB* gene clusters in VRE*fm* were carried by *de novo*-acquired variants of ICE Tn*1549*

The *vanB* clusters were carried on ICE Tn*1549* variants (Table 2 and Fig. S3) in all *vanB*-type VRE*fm* from 1996 and from 2010 to 2015. In the ST192-CT3/CT26 isolates that caused the largest outbreak affecting hospitals W1 (*n*=109) and W2 (*n*=4) during 2010–13, all but one isolate had an IS*L3* element integrated inside the *vanB* gene cluster in the intergenic region between the *vanS_B_* and *vanY_B_* genes (variant A in Fig. S3). All Tn*1549* in ST17, ST80 and ST203 were also larger than the prototype, mainly due to different IS element insertions (variants B, C and E in Fig. S3).

Acquisitions of Tn*1549* have been shown to occur *de novo* from anaerobic gut microbiota but Tn*1549* may also transfer between enterococci [68, 69]. Tn*1549* can move between enterococci as part of large chromosomal elements (90–250 kb), in which case the flanking region of Tn*1549* should be identical in the donor and recipient isolates [68, 70]. If Tn*1549* only transfers between or into enterococci, this should be associated with the transfer of a short coupling sequence from the donor into the recipient genome (5–6 bp) on either the left or right flank of Tn*1549* [71]. An identical prototypic Tn*1549* was found in one isolate of ST192-CT3/CT26 and one ST117-CT24 isolate that became the dominant clone in the same hospital from 2013. However, the prototypic Tn*1549* was integrated into different genomic locations with different flanking sequences in ST192-CT3/CT26 compared to ST117-CT24, suggesting independent ICE Tn*1549* acquisitions. While the VRE*fm* isolates of ST117-CT24 are mainly from one hospital in western Norway, the corresponding VSE isolates (*n*=21) were recovered from nine hospitals covering all four health regions. Thus, this VSE clone has been successful in spreading but likely picked up the *vanB* ICE Tn*1549* in hospital W1, as supported by the finding of a high prevalence of Tn*1549* in the non-enterococcal gut flora of admitted patients [72].

### *vanA* gene clusters are carried in unrelated CTs and by different plasmid variants with toxin–antitoxin systems

The *vanA* gene clusters were carried by different variants of Inc18 or RepA_N family plasmids across different CTs (Table 3 and Fig. S4). Briefly, in ST203-CT20 VRE*fm* a 55 kb *vanA* Inc18 plasmid with multiple IS integrations was identified. Mapping reads of *vanA-*type VRE*fm* isolates of this CT against the PacBio-sequenced ST203-CT20 isolate showed that 17 out of 19 *vanA* plasmids have 100 % coverage to our reference Inc18 plasmid. The *vanA* gene cluster in this Inc18 plasmid was not part of Tn*1546*, while other *vanA*-type clusters like those in ST80-CT3097, ST192-CT188 and ST202-CT3079 were carried by Tn*1546*. In the second largest cluster, ST80-CT3097, *vanA* was carried by a 32 kb RepA_N (rep17) plasmid. Other clusters showed *vanA* Inc18 and RepA_N variants of different sizes (Fig. S4 and Table 3).

Both *vanA* Inc18 and RepA_N plasmid types may confer increased fitness costs. The persistence of such plasmids has been linked to loss of phenotypic resistance, partial deletions, decreased copy number and toxin–antitoxin systems [73–75]. The partial homology and different sizes of the RepA_N *vanA*-containing plasmids in our study (Fig. S4) suggest significant rearrangements. Moreover, all the Norwegian VRE*fm vanA* RepA_N plasmids and the two *vanA* Inc18 plasmids encoded at least one putative toxin–antitoxin system, Axe–Txe and Epsilon–Zeta, respectively, supporting persistence (Table 3) [76, 77].

### Norwegian VRE*fm* and successful CTs have enriched virulomes compared to the more diverse VSE*fm* population

**Fig. 3.**
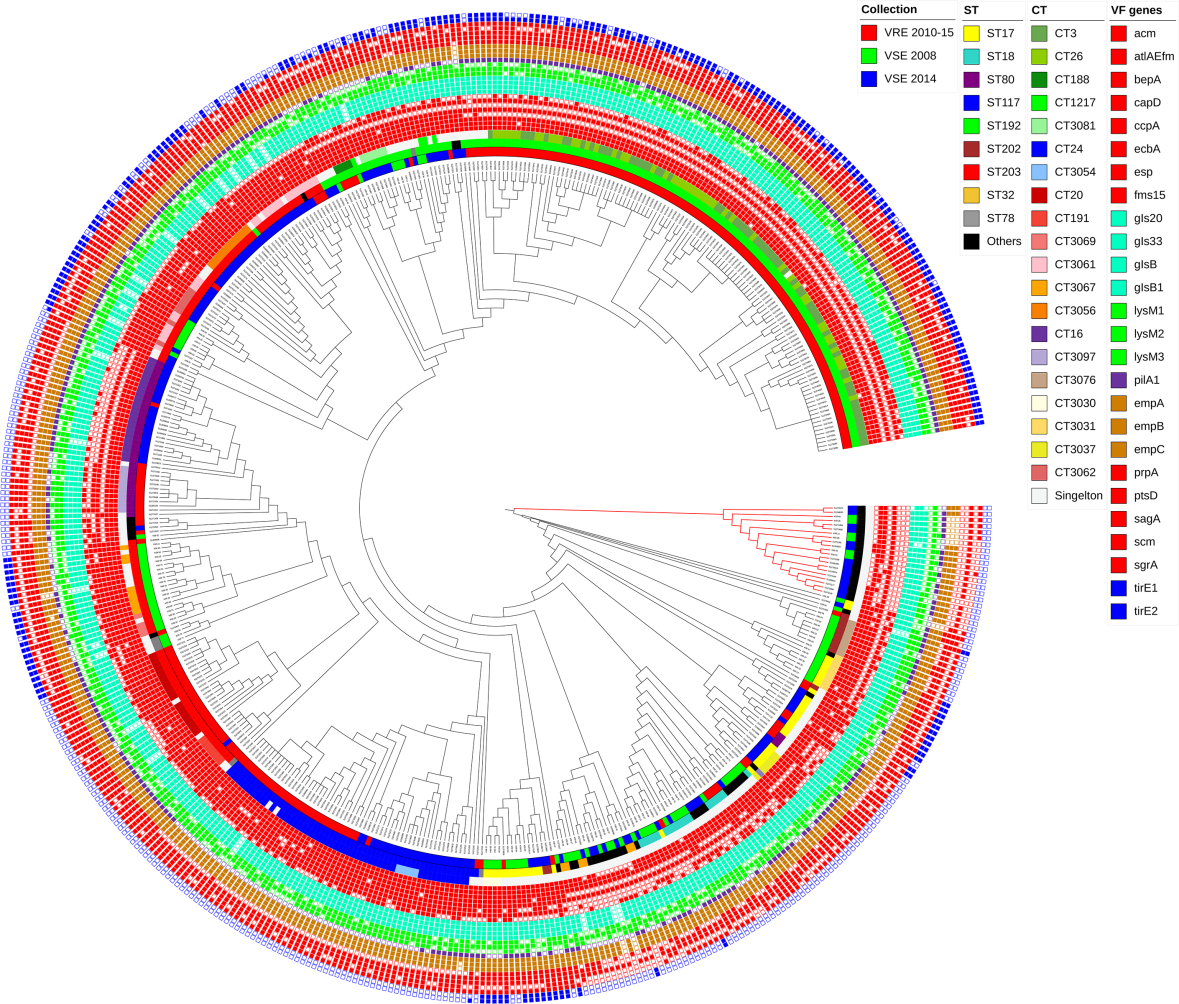
Core genome SNP tree of Norwegian *E. faecium* and *E. lactis* annotated with 26 virulence factor genes of *E. faecium *. Genes of one operon or some genes with similar functional categories are marked with the same colours. However, red is also used for genes that fall into dissimilar functional categories. All of the Norwegian isolates in this study were positive for *fnm* and *lysM4* and negative for *bonT/En* and *epx2*, which are not shown in the tree. Annotations shown from the inner layer are sample collection, ST, CT and one layer for each VF gene. The *E. lactis* clade is highlighted with red branches.

Fig. 3 illustrates the distribution of 26 out of 30 virulence determinants in the Norwegian *E. faecium *. The VF genes and their function are described in detail in File S2. blast analysis showed that all isolates were negative for *boNT*/En and *epx2* genes encoding exotoxins, which have only been reported in single isolates [53, 55], while positive for *fnm* and *lysM4*, which are not shown in the figure. The *acm*, *esp*, *pilA2, prpA*, *pstD*, *scm* and *srgA* genes are involved in colonization and biofilm formation [78–80], *tirEs* are associated with increased blood survival [60], and *gls* genes code for general stress proteins [52]. All of these genes are more prevalent in the Norwegian VRE*fm* than in the VSE*fm* (Table 4 and File S4). All VRE*fm* were positive for all the genes in the *empABC* operon coding for pilus subunits while for STs containing a mix of VRE and VSE isolates, some VSE lacked *empA* or *empB*, and in *E. lactis* 5/21 (24 %) of the isolates lacked the entire operon (Fig. 3 and File S4).

**Table 4.** Virulence factor (VF) genes and their distributions (%) in the Norwegian *E. faecium* and *E. lactis
*

VF gene	Percentage containing VF within major cluster types (CTs)							
	ST192-CT3/26	ST117-CT24	ST203-CT20	ST80-CT16	ST80-CT3097	Percentage with VF in all VRE*fm*	Percentage with VF in all *Efm*	Percentage with VF in all *E. lactis *
	(*n*=113)	(*n*=51)	(*n*=19)	(*n*=23)	(*n*=10)	(*n*=229)	(*n*=469)	(*n*=21)
	VRE	VRE/VSE	VRE	VRE/VSE	VRE	VRE	VRE/VSE	VSE
*atlA*_*Efm*_	99	98	100	100	100	99	99	100
*bepA*	100	100	100	100	100	100	100	71
*ccpA*	100	98	100	100	100	99	99	100
*empA*	100	100	100	100	100	100	99	76
*empB*	100	98	100	100	100	100	98	76
*empC*	100	98	100	100	100	100	99	76
*sgrA*	100	100	100	100	100	99	94	33
*fnm*	100	100	100	100	100	100	100	100
*ptsD*	99	100	100	100	100	99	97	0
*sagA*	100	100	100	100	90	99	99	95
*gls20*	100	100	94	91	100	98	95	95
*gls33*	100	100	94	91	100	98	95	95
*glsB*	100	100	94	91	100	98	95	95
*glsB1*	100	100	94	91	100	98	96	95
*lysM1*	71	68	89	69	90	73	72	24
*lysM2*	100	100	100	100	100	100	99	95
*lysM3*	84	86	42	43	50	47	46	24
*lysM4*	100	100	100	100	100	99	100	100
*acm*	97	100	100	100	100	99	97	28
*ecbA*	0	100	100	0	100	37	42	0
*fms15*	31	45	15	34	70	33	35	0
*pilA2*	99	13	89	100	50	78	67	66
*scm*	61	72	94	65	50	63	53	0
*esp*	99	100	68	0	10	90	83	0
*capD*	0	80	94	0	0	31	50	9
*prpA*	100	0	100	0	0	74	68	0
*tirE1*	94	0	0	0	0	54	47	0
*tirE2*	83	0	0	0	0	48	43	0
*bonT/En*	0	0	0	0	0	0	0	0
*epx2*	0	0	0	0	0	0	0	0

The successful VRE*fm* CTs (ST192-CT3/26, ST117-CT24, ST203-CT20, and ST80-CT3097) generally have a high but slightly variable number of virulence determinants (Fig. 3). ST192-CT3/26 (*n*=113) carries more VFs, in contrast to the ST80-CT16 cluster (containing only 1 VRE out of 23 isolates) lacking 8 VFs (*capD*, *ecbA*, *esp*, *prpA*, *tirE1*, *tirE2*, *boNT*/*En* and *epx2*). Many isolates in the latter cluster also lack *fms15*, *lysM1*, *lysM3* and *scm* (Fig. 3 and Table 4).

CT-specific VF profiles were generally observed regardless of the presence or absence of a *van* gene cluster. Interestingly, clinical VSE*fm* isolates may have fewer VFs compared to VRE*fm* isolates belonging to the same CT, and npST isolates have fewer VF genes than the predominant STs (see ST203-CT3061 Fig. 3). However, since the virulome of mixed VRE-/VSE*fm* clusters was highly variable, it was impossible to confirm the significance of the differences statistically.

### VRE*fs* incidence is much lower than VRE*fm*

Only 5 % of the Norwegian VRE 2010–15 isolates were VRE*fs*, an observation also found in a previous VRE study [81]. The VRE*fs* isolates (*n*=12), all *vanB*-type, clustered in ST6 (*n*=10) and ST28 (*n*=2). Nine of those formed three CTs, ST6-CT107 (*n*=4), ST6-CT1160 (*n*=3) and ST28-CT1162 (*n*=2) (Fig. S5). ST6 and ST28 are prevalent clinical STs of *E. faecalis* [82]. Ampicillin and linezolid resistance were not observed in VRE*fs*, but 8 out of 12 expressed HLGR (Table 1). The VRE*fs* are mainly associated with *vanB2*-Tn*1549* (*n*=8). However, in ST6-CT107 VRE*fs* (*n*=4), a *vanB1*-pTEF1 plasmid remnant was chromosomally integrated (Table 2 and Fig. S3) with 100 % identity and coverage to the typical *vanB1*-type VRE isolates of V583 (AE016830.1) [83]. The integrated *vanB1*-pTEF1 plasmid was also found in the genomes of two other V583 derivative isolates [84] and an isolate from the Netherlands (LR961935.1). The VRE*fm* versus VRE*fs* ratio indicates that *E. faecium* is more prone to acquire and maintain vancomycin resistance. Indeed, transfer of *vanB* Tn*1549* has been shown experimentally to occur from anaerobes to *E. faecium* [71], while Tn*1549* has not been shown to transfer on its own between enterococci and is only occasionally integrated into plasmids. This is one explanation for the low number of VRE*fs* in a low-prevalence setting where *vanB* is the dominant genotype.

### Trends in antimicrobial susceptibility patterns in *E. faecium*

The ampicillin resistance rate in the Norwegian invasive VSE*fm* isolates increased from 88 % in 2008 to 94 % in 2014, while it was 99.5 % in VRE*fm* during 2010–15. HLGR on the other hand, showed slightly decreasing prevalence in VSE*fm*, declining from 59 % in 2008 to 41 % in 2014 and an even lower rate (36 %) in VRE*fm* during 2010–15 (Table 1). Linezolid resistance (chromosomal) was only observed in two single isolates in this study. In comparison, the ampicillin and high-level gentamicin resistance rates in VRE*fm* blood culture isolates from EU/EEA 2012–18 were 99 and 49 %, respectively, in comparison to 89 and 43 %, respectively, for VSE*fm* [85].

### *E. lactis* is less resistant and has fewer known VFs than *E. faecium*

Since the *E. lactis* isolates (*n*=21) were identified as *E. faecium* by MALDI-TOF MS, they were also included in the phylogenetic tree of Norwegian *E. faecium* (*n*=490) (Fig. S6). In the earlier *E. faecium* classification, clade A is mainly formed by globally dominant STs, and no clear separation within clade A (A1 and A2 subclades) was observed. Boundaries for subclades in clade A are controversial in *E. faecium* population structure analysis [4] and may be affected by geographical context. For instance, in VRE*fm* isolates from Latin America, further subclading of A1 was proposed [86]. Thus, we refrain from specifying subclades in our collection (Fig. S6).

Globally, clade A isolates have been shown to be more prone to acquire genes, including resistance genes, while *E. lactis* (clade B) isolates are usually susceptible [4, 6]. All of the *E. lactis* (*n*=21) isolates in this study were npST VSE from 2008 and 2014 (black colour in ST ring of Figs 3 and S6). Our findings highlight significant differences in pheno- and genotype between *E. lactis* (clade B) and *E. faecium* (clade A). *E. lactis* isolates were found to be predominantly susceptible to vancomycin (Fig. S6) and aminoglycosides, whereas resistance to ampicillin and linezolid was limited (Table 1 and File S1). Vancomycin-resistant *E. lactis* isolates are rarely reported, although *E. lactis vanN*-type VRE have been observed in Japan (ST669) and the USA (ST240) [87]. Moreover, a lower number of VFs was typical for the *E. lactis* isolates (*n*=21), lacking from 13 to 19 of the investigated VF genes. None of the *E. lactis* isolates were shown to harbour *ecbA*, *esp*, *fms15*, *prpA*, *ptsD*, *scm*, *tirE1*, *tirE2*, *boNT*/En, or *epx2*, and *capD*, *lysM1*, *lysM3* and *sgrA* were found in a minority of isolates (Table 4, Fig. 3 and File S4). Our results reveal differences in *E. lactis* VF profiles compared to others using a different VF database [88]. Notably, while *scm* was lacking in Norwegian *E. lactis* (*n*=21), it was present in four of nine *E. lactis* in the study by Roer *et al*. [88]. Potentially significant differences in prevalence were also observed for *srgA* and *bepA*. However, the small number of *E. lactis* isolates in both studies does not support an overall conclusion.

### Study strengths and limitations

The main issues in the global molecular epidemiology of enterococci are the bias caused by (i) the skewed geographical representation and (ii) the dominance of VRE. Most of the examined VRE and VSE genomes are submitted from Europe, followed by Japan, Australia and the USA. Thus, the epidemiology of VRE is less known in other parts of the world (Africa, the Middle East and South Asia). Moreover, most of the studies are biased by an overrepresentation of antibiotic-resistant outbreak isolates. In this study, the sample selection of VRE was performed randomly across time and region, including different types of infection sources and carriers. In addition, VSE isolates were included for genomic comparison. Thus, the current strain collection is more representative of the concomitant VSE and VRE in a defined setting, a low-prevalence AMR European context.

In the global trees and genomic comparisons, we used the complete closed genomes of the *E. faecium* (*n*=272 as of 11 May 2022), which included only 2 % of the *E. faecium* genomes (all assembly levels) available in the NCBI [89]. Excluding 98 % of the genomes, as well as missing data from the rest of the world, may increase the risk of overlooking an association in the global population structure.

Other studies include several putative VFs in the *E. faecium* virulome [86, 90]. All VF genes included in our study are experimentally confirmed virulence determinants (references listed in File S2), and we believe this provides a more conservative and less speculative approach.

## Conclusions

Our study highlights that globally prevalent clones, and particularly concurrent European CTs, influence the population structure of both the vancomycin-resistant and -sensitive Norwegian *E. faecium *. The prevalent Norwegian VRE*fm* CTs have acquired more virulence determinants than the more diverse nationwide VSE*fm* population. The majority of the VRE*fm* isolates were *vanB* type, likely driven by outbreaks in the healthcare setting but also formed by *de novo* acquisition of *vanB* from the gut microbiota. VRE*fs* are much rarer than VRE*fm* and are all *vanB* type.

## Supplementary Data

Supplementary material 1

Supplementary material 2

## References

[1] Fiore E, Van Tyne D, Gilmore MS (2019). Pathogenicity of enterococci. Microbiol Spectr.

[2] Ramos S, Silva V, Dapkevicius M de LE, Igrejas G, Poeta P (2020). Enterococci, from harmless bacteria to a pathogen. Microorganisms.

[3] García-Solache M, Rice LB (2019). The enterococcus: a model of adaptability to its environment. Clin Microbiol Rev.

[4] van Hal SJ, Willems RJL, Gouliouris T, Ballard SA, Coque TM (2022). The interplay between community and hospital *Enterococcus faecium* clones within health-care settings: a genomic analysis. Lancet Microbe.

[5] Arredondo-Alonso S, Top J, McNally A, Puranen S, Pesonen M (2020). Plasmids shaped the recent emergence of the major nosocomial pathogen *Enterococcus faecium*. mBio.

[6] Lebreton F, van Schaik W, McGuire AM, Godfrey P, Griggs A (2013). Emergence of epidemic multidrug-resistant *Enterococcus faecium* from animal and commensal strains. mBio.

[7] Belloso Daza MV, Cortimiglia C, Bassi D, Cocconcelli PS (2021). Genome-based studies indicate that the *Enterococcus faecium* Clade B strains belong to *Enterococcus lactis* species and lack of the hospital infection associated markers. Int J Syst Evol Microbiol.

[8] Sparo M, Delpech G, García Allende N (2018). Impact on public health of the spread of high-level resistance to gentamicin and vancomycin in enterococci. Front Microbiol.

[9] Xavier BB, Coppens J, De Koster S, Rajakani SG, Van Goethem S (2021). Novel vancomycin resistance gene cluster in *Enterococcus faecium* ST1486, Belgium, June 2021. Euro Surveill.

[10] Moura TM de, Cassenego APV, Campos FS, Ribeiro AML, Franco AC (2013). Detection of vanC1 gene transcription in vancomycin-susceptible *Enterococcus faecalis*. Mem Inst Oswaldo Cruz.

[11] Sivertsen A, Pedersen T, Larssen KW, Bergh K, Rønning TG (2016). A silenced *vanA* gene cluster on a transferable plasmid caused an outbreak of vancomycin-variable enterococci. Antimicrob Agents Chemother.

[12] Arredondo-Alonso S, Top J, Corander J, Willems RJL, Schürch AC (2021). Mode and dynamics of *vanA*-type vancomycin resistance dissemination in Dutch hospitals. Genome Med.

[13] Hegstad K, Mikalsen T, Coque TM, Werner G, Sundsfjord A (2010). Mobile genetic elements and their contribution to the emergence of antimicrobial resistant *Enterococcus faecalis* and *Enterococcus faecium*. Clin Microbiol Infect.

[14] Zhou X, Chlebowicz MA, Bathoorn E, Rosema S, Couto N (2018). Elucidating vancomycin-resistant *Enterococcus faecium* outbreaks: the role of clonal spread and movement of mobile genetic elements. J Antimicrob Chemother.

[15] Weiss RA (2002). Virulence and pathogenesis. Trends Microbiol.

[16] Mundy LM, Sahm DF, Gilmore M (2000). Relationships between enterococcal virulence and antimicrobial resistance. Clin Microbiol Rev.

[17] Gao W, Howden BP, Stinear TP (2018). Evolution of virulence in *Enterococcus faecium*, a hospital-adapted opportunistic pathogen. Curr Opin Microbiol.

[18] NORM/NORM-VET 2020 (2021). Usage of Antimicrobial Agents and Occurrence of Antimicrobial Resistance in Norway. ISSN:1502-2307 (Print) / 1890-9965 (Electronic).

[19] NORM/NORM-VET 2008 (2009). Usage of Antimicrobial Agents and Occurrence of Antimicrobial Resistance in Norway. ISSN: 1502-2307 (Print) / 1890-9965 (Electronic).

[20] NORM/NORM-VET 2014 (2015). Usage of Antimicrobial Agents and Occurrence of Antimicrobial Resistance in Norway. ISSN: 1502-2307 (Print) / 1890-9965 (Electronic). ISSN:1502-2307 (Electronic).

[21] Rosvoll TCS, Lindstad BL, Lunde TM, Hegstad K, Aasnaes B (2012). Increased high-level gentamicin resistance in invasive Enterococcus faecium is associated with aac(6´)Ie-aph(2″)Ia-encoding transferable megaplasmids hosted by major hospital-adapted lineages. FEMS Immunol Med Microbiol.

[22] Grimes DA, Schulz KF (2005). Compared to what? Finding controls for case-control studies. Lancet.

[23] Dahl KH, Røkenes TP, Lundblad EW, Sundsfjord A (2003). Nonconjugative transposition of the *vanB*-containing Tn*5382*-like element in *Enterococcus faecium*. Antimicrob Agents Chemother.

[24] European Committee on Antimicrobial Susceptibility Testing – EUCAST (2019). Antimicrobial susceptibility testing: EUCAST disk diffusion method, version 7.0. https://www.eucast.org/fileadmin/src/media/PDFs/EUCAST_files/Disk_test_documents/2019_manuals/Manual_v_7.0_EUCAST_Disk_Test_2019.pdf.

[25] European Committee on Antimicrobial Susceptibility Testing (2019). Breakpoint tables for interpretation of MICs and zone diameters, version 9.0, 2019. https://www.eucast.org/fileadmin/src/media/PDFs/EUCAST_files/Breakpoint_tables/v_9.0_Breakpoint_Tables.pdf.

[26] Swenson JM, Clark NC, Ferraro MJ, Sahm DF, Doern G (1994). Development of a standardized screening method for detection of vancomycin-resistant enterococci. J Clin Microbiol.

[27] AL Rubaye MTS, Janice J, Bjørnholt JV, Jakovljev A, Hultström ME (2021). Novel genomic islands and a new *vanD*-subtype in the first sporadic VanD-type vancomycin resistant enterococci in Norway. PLoS One.

[28] Bolger AM, Lohse M, Usadel B (2014). Trimmomatic: a flexible trimmer for Illumina sequence data. Bioinformatics.

[29] Andrews S (2010). Fastqc: a quality control tool for high throughput sequence data. Babraham Bioinform.

[30] Wick RR, Judd LM, Gorrie CL, Holt KE (2017). Unicycler: resolving bacterial genome assemblies from short and long sequencing reads. PLoS Comput Biol.

[31] Gurevich A, Saveliev V, Vyahhi N, Tesler G (2013). QUAST: quality assessment tool for genome assemblies. Bioinformatics.

[32] Koren S, Walenz BP, Berlin K, Miller JR, Bergman NH (2017). Canu: scalable and accurate long-read assembly via adaptive *k*-mer weighting and repeat separation. Genome Res.

[33] Walker BJ, Abeel T, Shea T, Priest M, Abouelliel A (2014). Pilon: an integrated tool for comprehensive microbial variant detection and genome assembly improvement. PLoS One.

[34] Hunt M, Silva ND, Otto TD, Parkhill J, Keane JA (2015). Circlator: automated circularization of genome assemblies using long sequencing reads. Genome Biol.

[35] Tatusova T, DiCuccio M, Badretdin A, Chetvernin V, Nawrocki EP (2016). NCBI prokaryotic genome annotation pipeline. Nucleic Acids Res.

[36] Seemann T (2015). Snippy, Rapid haploid variant calling and core genome alignment. https://github.com/tseemann/snippy.

[37] Jolley KA, Maiden MCJ (2010). BIGSdb: scalable analysis of bacterial genome variation at the population level. BMC Bioinformatics.

[38] de Been M, Pinholt M, Top J, Bletz S, Mellmann A (2015). Core genome multilocus sequence typing scheme for high-resolution typing of *Enterococcus faecium*. J Clin Microbiol.

[39] Neumann B, Prior K, Bender JK, Harmsen D, Klare I (2019). A core genome multilocus sequence typing scheme for *Enterococcus faecalis*. J Clin Microbiol.

[40] Jolley KA, Bray JE, Maiden MCJ (2018). Open-access bacterial population genomics: BIGSdb software, the PubMLST.org website and their applications. Wellcome Open Res.

[41] Treangen TJ, Ondov BD, Koren S, Phillippy AM (2014). The Harvest suite for rapid core-genome alignment and visualization of thousands of intraspecific microbial genomes. Genome Biol.

[42] Letunic I, Bork P (2021). Interactive Tree Of Life (iTOL) v5: an online tool for phylogenetic tree display and annotation. Nucleic Acids Res.

[43] Seemann T Abricate,Github. https://github.com/tseemann/abricate.

[44] Darling ACE, Mau B, Blattner FR, Perna NT (2004). Mauve: multiple alignment of conserved genomic sequence with rearrangements. Genome Res.

[45] Altschul SF, Gish W, Miller W, Myers EW, Lipman DJ (1990). Basic local alignment search tool. J Mol Biol.

[46] Carver TJ, Rutherford KM, Berriman M, Rajandream M-A, Barrell BG (2005). ACT: the Artemis comparison tool. Bioinformatics.

[47] Sullivan MJ, Petty NK, Beatson SA (2011). Easyfig: a genome comparison visualizer. Bioinformatics.

[48] Robertson J, Nash JHE (2018). MOB-suite: software tools for clustering, reconstruction and typing of plasmids from draft assemblies. Microb Genom.

[49] Li H (2013). Aligning sequence reads, clone sequences and assembly Contigs with BWA-MEM. arXiv.

[50] Danecek P, Bonfield JK, Liddle J, Marshall J, Ohan V (2021). Twelve years of SAMtools and BCFtools. Gigascience.

[51] Somarajan SR, La Rosa SL, Singh KV, Roh JH, Höök M (2015). The fibronectin-binding protein Fnm contributes to adherence to extracellular matrix components and virulence of *Enterococcus faecium*. Infect Immun.

[52] Choudhury T, Singh KV, Sillanpää J, Nallapareddy SR, Murray BE (2011). Importance of two *Enterococcus faecium* loci encoding Gls-like proteins for in vitro bile salts stress response and virulence. J Infect Dis.

[53] Xiong X, Tian S, Yang P, Lebreton F, Bao H (2022). Emerging Enterococcus pore-forming toxins with MHC/HLA-I as receptors. Cell.

[54] Paganelli FL, Willems RJL, Jansen P, Hendrickx A, Zhang X (2013). *Enterococcus faecium* biofilm formation: identification of major autolysin AtlAEfm, associated Acm surface localization, and AtlAEfm-independent extracellular DNA Release. mBio.

[55] Zhang S, Lebreton F, Mansfield MJ, Miyashita S-I, Zhang J (2018). Identification of a botulinum neurotoxin-like toxin in a commensal strain of *Enterococcus faecium*. Cell Host Microbe.

[56] Somarajan SR, Roh JH, Singh KV, Weinstock GM, Murray BE (2014). CcpA is important for growth and virulence of *Enterococcus faecium*. Infect Immun.

[57] Cacaci M, Giraud C, Leger L, Torelli R, Martini C (2018). Expression profiling in a mammalian host reveals the strong induction of genes encoding LysM domain-containing proteins in *Enterococcus faecium*. Sci Rep.

[58] Ali L, Blum HE, Sakιnç T (2019). Detection and characterization of bacterial polysaccharides in drug-resistant enterococci. Glycoconj J.

[59] Zhang X, Top J, de Been M, Bierschenk D, Rogers M (2013). Identification of a genetic determinant in clinical *Enterococcus faecium* strains that contributes to intestinal colonization during antibiotic treatment. J Infect Dis.

[60] Wagner TM, Janice J, Paganelli FL, Willems RJ, Askarian F (2018). *Enterococcus faecium* TIR-domain genes are part of a gene cluster which promotes bacterial survival in blood. Int J Microbiol.

[61] Palmer KL, Schaik W, Willems RJL, Gilmore MS, Gilmore MS, Clewell DB, Ike Y (2014). Enterococci: From Commensals to Leading Causes of Drug Resistant Infection.

[62] NORM/NORM-VET 2019 (2020). Usage of Antimicrobial Agents and Occurrence of Antimicrobial Resistance in Norway. ISSN:1502-2307 (Print) / 1890-9965 (Electronic).

[63] Werner G, Neumann B, Weber RE, Kresken M, Wendt C (2020). Thirty years of VRE in Germany - “expect the unexpected”: The view from the National Reference Centre for Staphylococci and Enterococci. Drug Resist Updat.

[64] Zhou W, Zhou H, Sun Y, Gao S, Zhang Y (2020). Characterization of clinical enterococci isolates, focusing on the vancomycin-resistant enterococci in a tertiary hospital in China: based on the data from 2013 to 2018. BMC Infect Dis.

[65] Sivertsen A, Billström H, Melefors Ö, Liljequist BO, Wisell KT (2014). A multicentre hospital outbreak in Sweden caused by introduction of a *vanB2* transposon into a stably maintained pRUM-plasmid in an *Enterococcus faecium* ST192 clone. PLoS One.

[66] Weber A, Maechler F, Schwab F, Gastmeier P, Kola A (2020). Increase of vancomycin-resistant *Enterococcus faecium* strain type ST117 CT71 at Charité - Universitätsmedizin Berlin, 2008 to 2018. Antimicrob Resist Infect Control.

[67] Hammerum AM, Justesen US, Pinholt M, Roer L, Kaya H (2019). Surveillance of vancomycin-resistant enterococci reveals shift in dominating clones and national spread of a vancomycin-variable *vanA Enterococcus faecium* ST1421-CT1134 clone, Denmark, 2015 to March 2019. Euro Surveill.

[68] Bender JK, Kalmbach A, Fleige C, Klare I, Fuchs S (2016). Population structure and acquisition of the *vanB* resistance determinant in German clinical isolates of *Enterococcus faecium* ST192. Sci Rep.

[69] Howden BP, Holt KE, Lam MMC, Seemann T, Ballard S (2013). Genomic insights to control the emergence of vancomycin-resistant enterococci. mBio.

[70] Quintiliani R, Courvalin P (1994). Conjugal transfer of the vancomycin resistance determinant *vanB* between enterococci involves the movement of large genetic elements from chromosome to chromosome. FEMS Microbiol Lett.

[71] Launay A, Ballard SA, Johnson PDR, Grayson ML, Lambert T (2006). Transfer of vancomycin resistance transposon Tn*1549* from *Clostridium symbiosum* to *Enterococcus* spp. in the gut of gnotobiotic mice. Antimicrob Agents Chemother.

[72] Nygaard RM, Hegstad K, Kommedal Ø, Lindemann PC (2019). 12th International Meeting on Microbial Epidemiological Markers.

[73] Tedim AP, Lanza VF, Rodríguez CM, Freitas AR, Novais C (2021). Fitness cost of vancomycin-resistant *Enterococcus faecium* plasmids associated with hospital infection outbreaks. J Antimicrob Chemother.

[74] Wagner TM, Janice J, Sivertsen A, Sjögren I, Sundsfjord A (2021). Alternative *vanHAX* promoters and increased *vanA*-plasmid copy number resurrect silenced glycopeptide resistance in *Enterococcus faecium*. J Antimicrob Chemother.

[75] Freitas AR, Tedim AP, Francia MV, Jensen LB, Novais C (2016). Multilevel population genetic analysis of *vanA* and *vanB Enterococcus faecium* causing nosocomial outbreaks in 27 countries (1986-2012). J Antimicrob Chemother.

[76] Grady R, Hayes F (2003). Axe-Txe, a broad-spectrum proteic toxin-antitoxin system specified by a multidrug-resistant, clinical isolate of *Enterococcus faecium*. Mol Microbiol.

[77] Rosvoll TCS, Pedersen T, Sletvold H, Johnsen PJ, Sollid JE (2010). PCR-based plasmid typing in *Enterococcus faecium* strains reveals widely distributed pRE25-, pRUM-, pIP501- and pHTbeta-related replicons associated with glycopeptide resistance and stabilizing toxin-antitoxin systems. FEMS Immunol Med Microbiol.

[78] Heikens E, Bonten MJM, Willems RJL (2007). Enterococcal surface protein Esp is important for biofilm formation of *Enterococcus faecium* E1162. J Bacteriol.

[79] Wagner T, Joshi B, Janice J, Askarian F, Škalko-Basnet N (2018). *Enterococcus faecium* produces membrane vesicles containing virulence factors and antimicrobial resistance related proteins. J Proteomics.

[80] Revtovich AV, Tjahjono E, Singh KV, Hanson BM, Murray BE (2021). Development and characterization of high-throughput *Caenorhabditis elegans* - *Enterococcus faecium* infection model. Front Cell Infect Microbiol.

[81] Elstrøm P, Astrup E, Hegstad K, Samuelsen Ø, Enger H (2019). The fight to keep resistance at bay, epidemiology of carbapenemase producing organisms (CPOs), vancomycin resistant enterococci (VRE) and methicillin resistant *Staphylococcus aureus* (MRSA) in Norway, 2006 - 2017. PLoS One.

[82] Pöntinen AK, Top J, Arredondo-Alonso S, Tonkin-Hill G, Freitas AR (2021). Apparent nosocomial adaptation of *Enterococcus faecalis* predates the modern hospital era. Nat Commun.

[83] Paulsen IT, Banerjei L, Myers GSA, Nelson KE, Seshadri R (2003). Role of mobile DNA in the evolution of vancomycin-resistant *Enterococcus faecalis*. Science.

[84] Furlan S, Matos RC, Kennedy SP, Doublet B, Serror P (2022). Fitness restoration of a genetically tractable *Enterococcus faecalis* V583 derivative to study decoration-related phenotypes of the enterococcal polysaccharide antigen. mSphere.

[85] Ayobami O, Willrich N, Reuss A, Eckmanns T, Markwart R (2020). The ongoing challenge of vancomycin-resistant *Enterococcus faecium* and *Enterococcus faecalis* in Europe: an epidemiological analysis of bloodstream infections. Emerg Microbes Infect.

[86] Rios R, Reyes J, Carvajal LP, Rincon S, Panesso D (2020). Genomic epidemiology of vancomycin-resistant *Enterococcus faecium* (VRE*fm*) in Latin America: revisiting the global VRE population structure. Sci Rep.

[87] Lebreton F, Valentino MD, Schaufler K, Earl AM, Cattoir V (2018). Transferable vancomycin resistance in clade B commensal-type *Enterococcus faecium*. J Antimicrob Chemother.

[88] Roer L, Kaya H, Tedim AP, Novais C, Coque TM (2023). 33rd European Congress of Clinical Microbiology and Infectious Diseases.

[89] NCBI *Enterococcus faecium* assemblies. https://www.ncbi.nlm.nih.gov/assembly/?term=enterococcus%20faecium.

[90] van Hal SJ, Willems RJL, Gouliouris T, Ballard SA, Coque TM (2021). The global dissemination of hospital clones of *Enterococcus faecium*. Genome Med.

