# Supplementary material for: The population structure of vancomycin-resistant and -susceptible Enterococcus faecium in a low-prevalence antimicrobial resistance setting is highly influenced by circulating global hospital-associated clones
Source: Microb Genom. 2023 Dec 19;9(12):001160. doi: 10.1099/mgen.0.001160 (PMC10763505; doi:10.1099/mgen.0.001160)
Supplement: Supplementary material 1 [file mgen-9-1160-s001.pdf]

SUPPLEMENTARY FIGURES, FILES S2 AND S3, AND DOCUMENT S1

The population structure of vancomycin-resistant and -susceptible *Enterococcus faecium* in a low-prevalence antimicrobial resistance setting is highly influenced by circulating global hospital-associated clones

Mushtaq AL Rubaye, Jessin Janice, Jørgen Vildershøj Bjørnholt, Oliver Kacelnik, Bjørg C. Haldorsen, Randi M. Nygaard, Joachim Hegstad, Arnfinn Sundsfjord, Kristin Hegstad and the Norwegian VRE study group.

SUPPLEMENTARY FIGURES

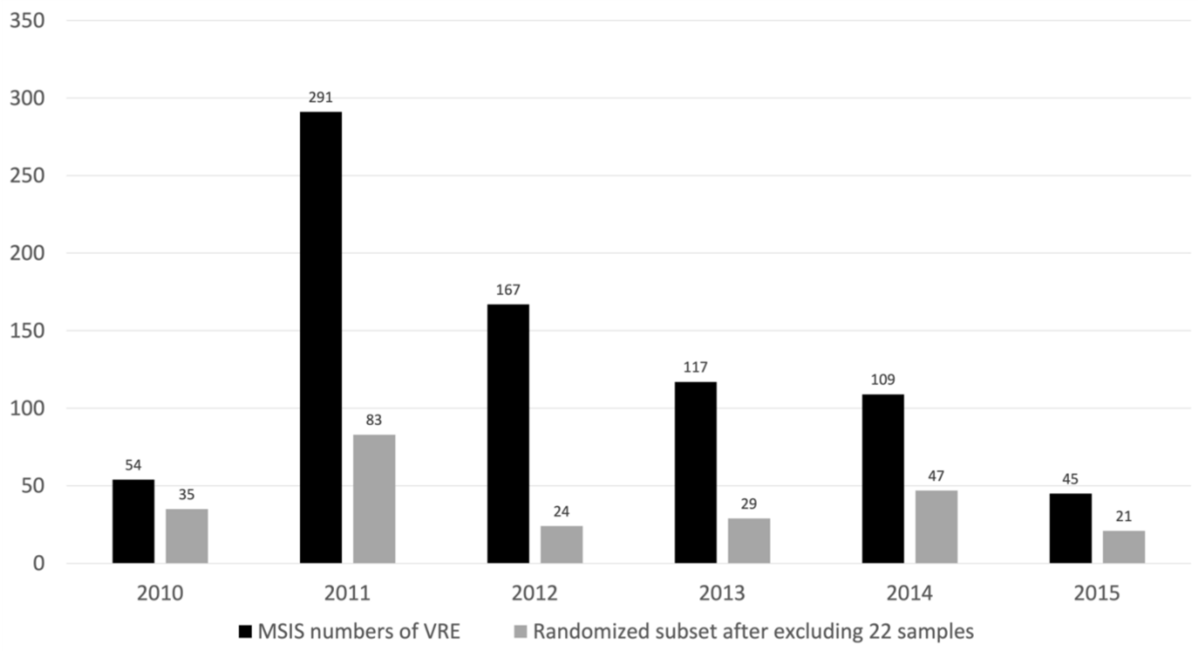

Fig. S1. Norwegian vancomycin resistant enterococci (VRE) total numbers per year accoring to the Norwegian Surveillance System for Communicable Diseases (MSIS) versus the number of VRE included in this study.

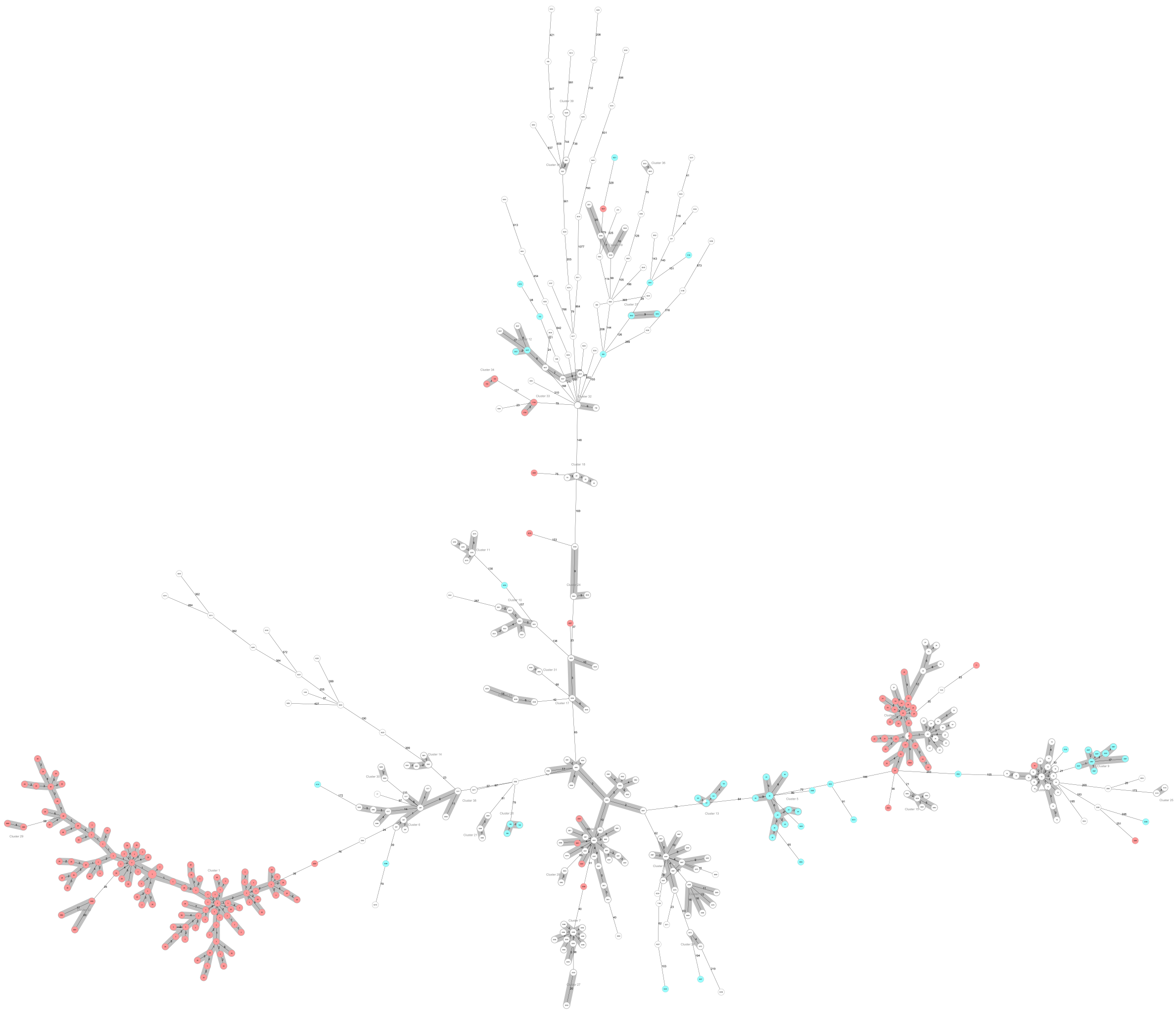

Fig. S2. Minimum spanning tree of the 490 Norwegian *E. faecium* and *E. lactis* isolates from the VSE and VRE 2010-2015 collections based on the core genome (cg)MLST target gene scheme. Samples are double-labelled (*van*-type and cluster type) and the colour of the leaves is based on *van*-type. In each cluster, the isolates are connected by a grey area. The number of allelic differences is shown next to the black lines.

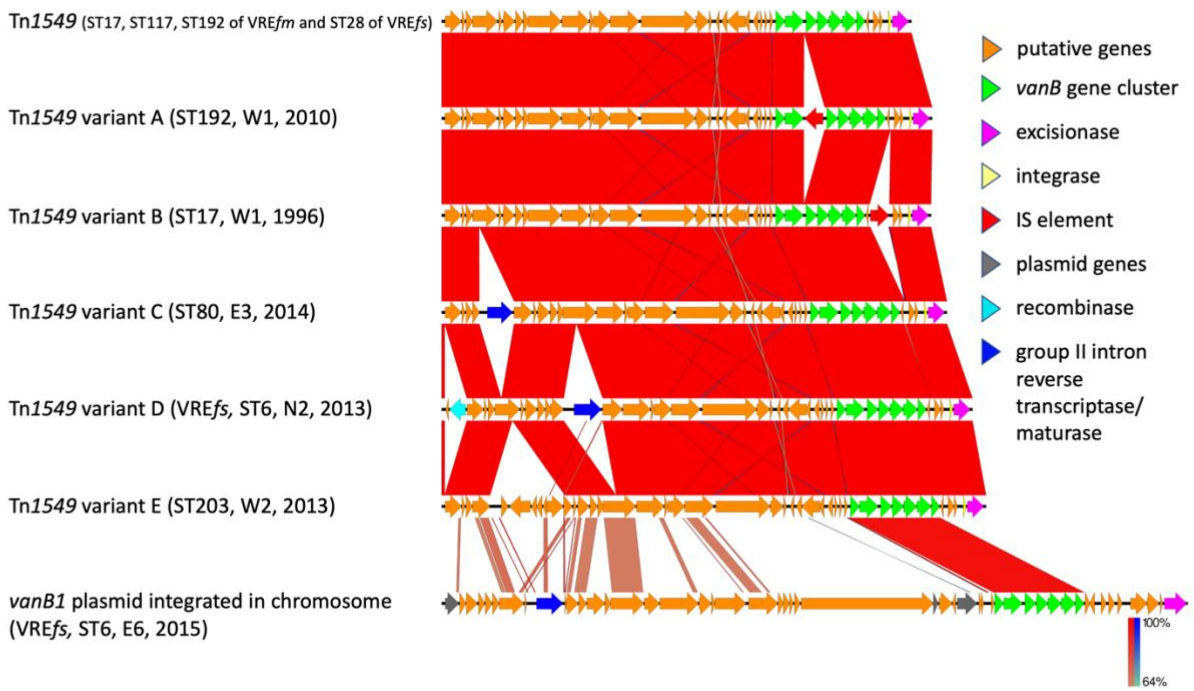

Fig. S3. Comparison of the mobile genetic elements (MGEs) harbouring a *vanB* gene cluster. The red and blue gradient bars represent direct and reverted sequence matches, respectively. One representative from each variant of MGE harbouring *vanB* gene cluster are shown in the Figure. Arrows symbolise the coding sequences and indicate the direction of transcription. Genes with different functions are shown in different colours according to the legend beside the figure.

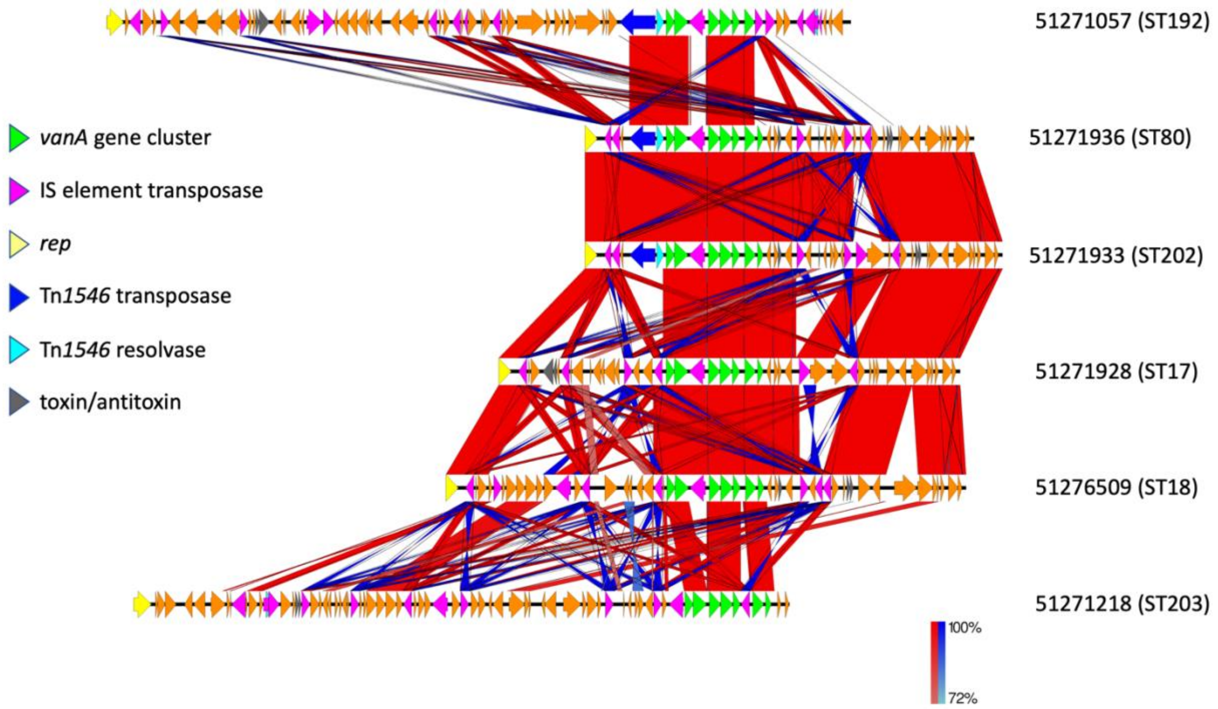

Fig. S4. Comparison of *E. faecium* plasmids carrying the *vanA* gene cluster. Red shows the direct and blue the inverted sequence matches. In ST80, ST202, and ST192, the *vanA* gene cluster is carried by Tn*1546* integrated in the plasmids. Genes with different functions are shown in different colours according to the legend beside the figure. The Tn*1546* transposase gene in ST192 is larger than the transposase gene in ST202 and ST80 by 268 amino acids. We don’t know if this affects the transposase functionality.

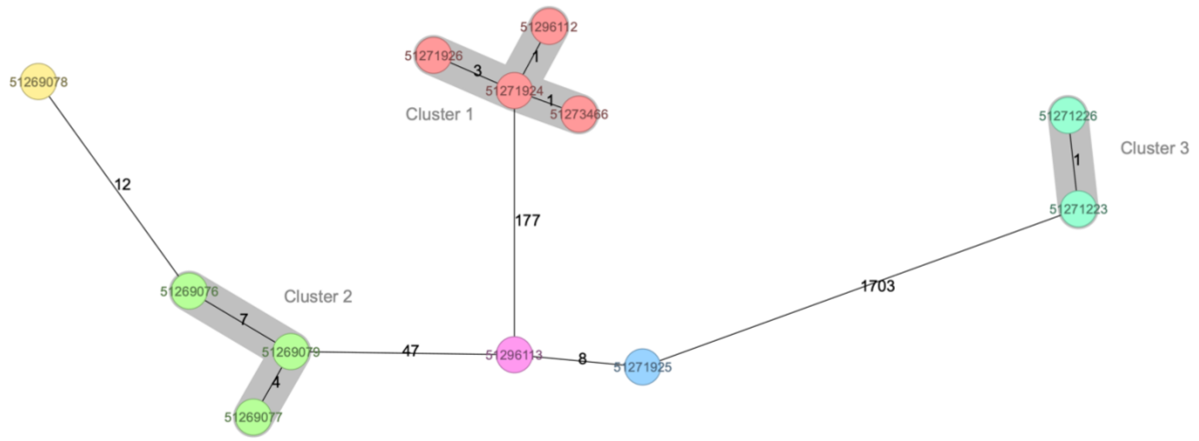

Fig S5. Minimum spanning tree of the 12 Norwegian *E. faecalis vanB*-type VRE isolates based on the cgMLST target gene scheme. A grey area connects isolates belonging to the same cluster. The number of allelic differences is shown next to the black lines. Different sequence types (STs) have different colour code.

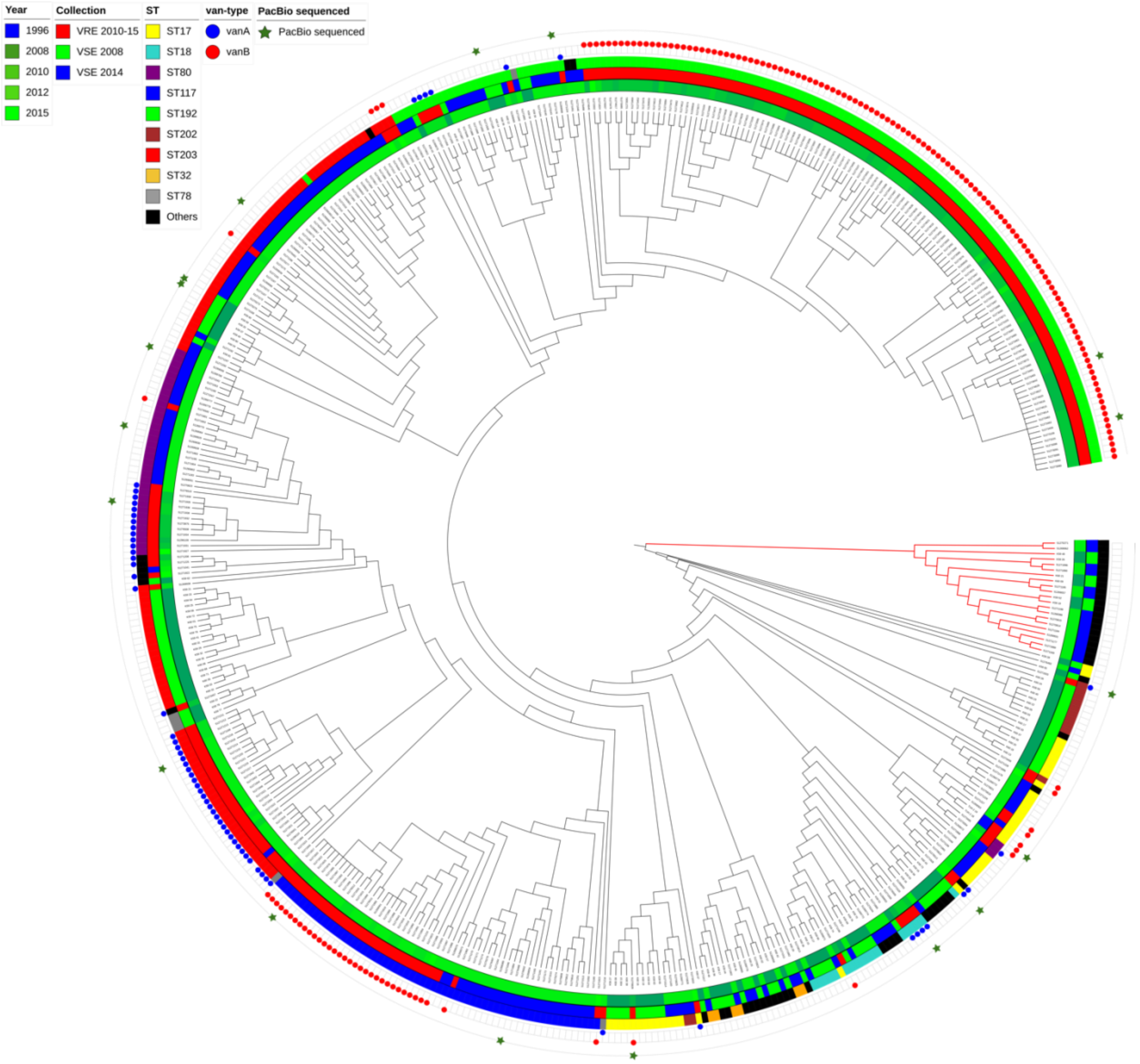

Fig. S6. Norwegian *E. faecium* and *E. lactis* core genome SNP tree. Metadata added from the inner layer are year of isolation, sample collection, ST, *van*-type (*vanA* or *vanB*), and information about which isolates were sequenced by long read (PacBio) technology. The nine most prevalent STs are highlighted in different colours, while all non-prevalent STs are marked in black. *E. lactis* isolates are included in this tree and their clade is highlighted with red-coloured branches.

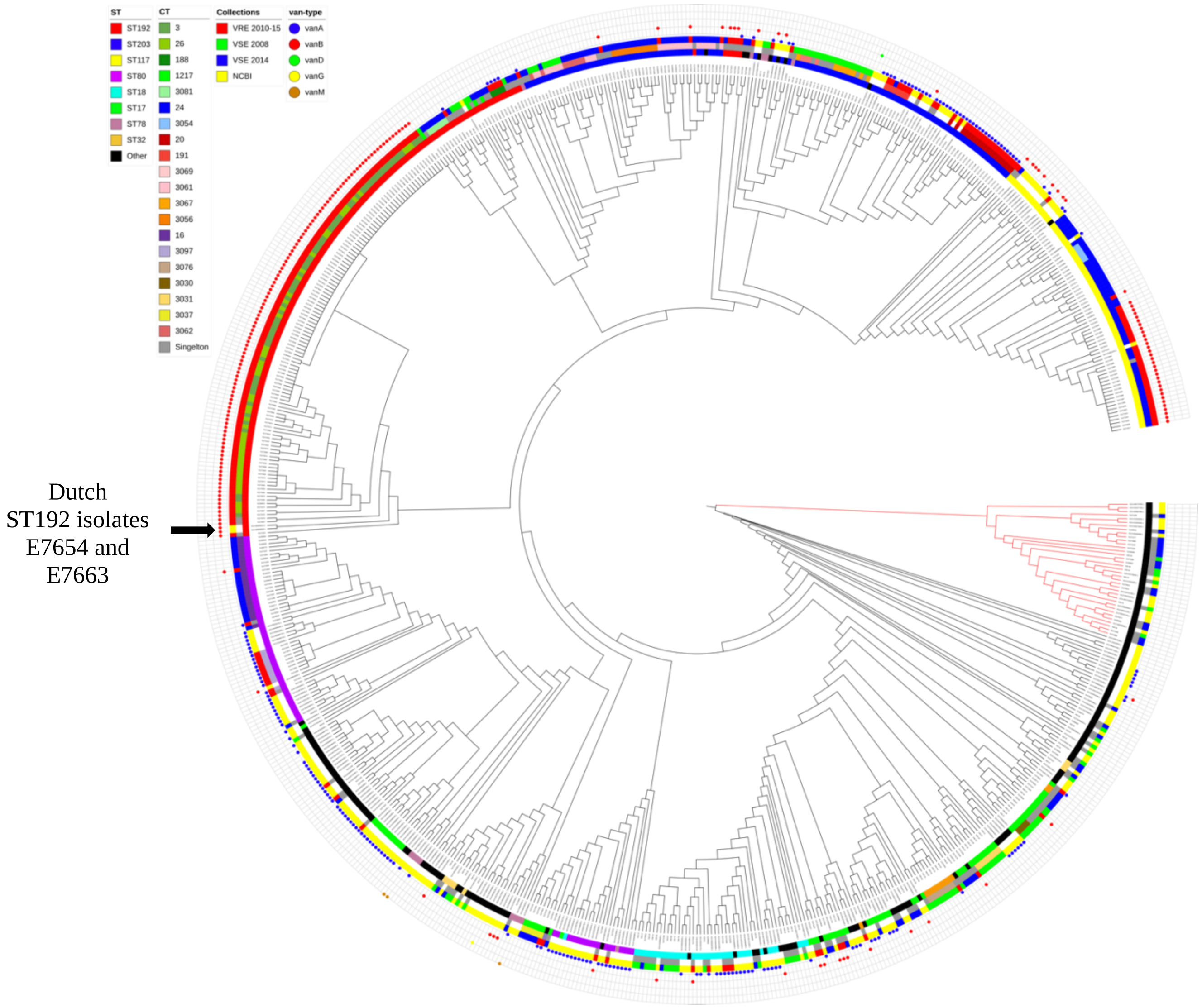

Fig. S7. Global *E. faecium* core genome SNP tree. The midpoint rooted tree includes all complete *E. faecium* assemblies from NCBI (*n*=272) as of 11.05.2022 in addition to the Norwegian *E. faecium* and *E. lacits* isolates of our study (*n*=490). Annotations shown from the inner layer are ST, CT, sample collection, and *van*-type. The most prevalent STs and CTs are highlighted in different colours. All non-prevalent STs and CTs are marked in black and grey, respectively. *E. lactis* clade is highlighted with red-coloured branches.

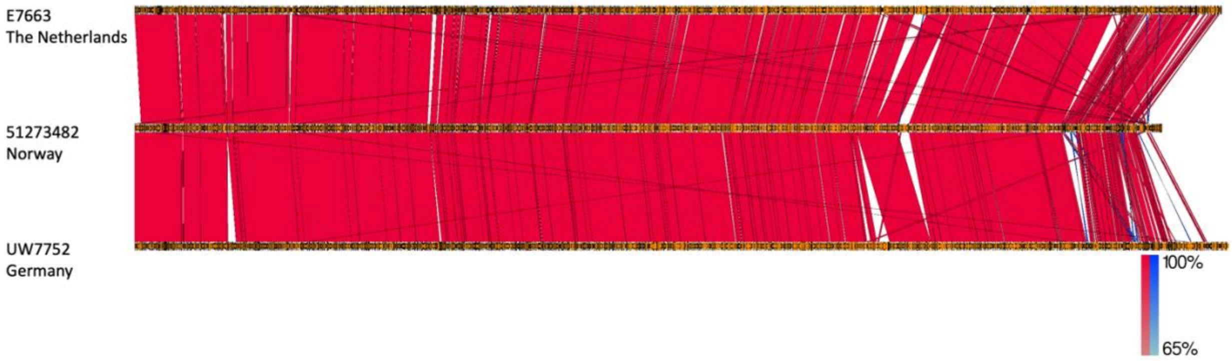

Fig. S8. Comparison of the genetically closely related *vanB*-type ST192-CT3 VRE*fm* isolates from Norway, Germany, and the Netherlands. Direct and inverted sequence matches are represented by red and blue gradient bars, respectively.

SUPPLEMENTARY FILES

File S2. Table with 30 experimentally confirmed virulence factor (VF) genes in *E. faecium* including a short description of the VFs and the accession number of the sequence that was used to build our VF database.

	**Virulence factor genes**	**Alternative gene names**	**DNA tag**	**Function**	**Reference**
1	*atlA*$_{Efm}$		CP003583.1:c2230282-2228117	Major autolysin, biofilm formation	1
2	*acm*	*fms8*	ABQJ01000138.1:27210-29228	Collagen binding adhesin, MSCRAMM, similar to Ace in *E. faecalis*	2, 17
3	*bepA*	*fruA*	ABQJ01000011.1 :42962-44380	Biofilm and endocarditis-associated permease A (PTS associated)	4
4	*boNT*/En		NGLI01000004.1:167397-171236	Botulinum neurotoxin-like toxin	20
5	*capD*		CP003583.1:892837-893844	Capsular polysaccharide biosynthesis protein, adhesion, avoid opsonic killing	9, 19
6	*ccpA*		AEBU01000039.1:1370-2389	Catabolite control protein A, growth, virulence	3
7	*ecbA*	*orf2430*	ABQJ01000112.1 :5675-8902	Collagen binding MSCRAMMM, adhesion	8
8	*empABC*	*empA* (pilin) previously *ebpA*$_{fm}$ and *fms1*	AAAK03000002.1 :97025-100414	Pili, biofilm formation (mainly EmpA) and adherence to ECM proteins (EmpAB), reduced virulence in murine UTI (EmpABC) and infective endocarditis (EmpA) models	22, 11, 6
9		*empB* (pilin) previously *ebpB*$_{fm}$ and *fms5*	AAAK03000002.1 :95601-97022		
10		*empC* (major pilus subunit) previously *ebpC*$_{fm}$, *pilB* and *fms9*	AAAK03000002.1 :93727-95604		
11	*epx2*		LGAN01000048.1:c51744-50740	Cytotoxic pore-forming toxin, preferred receptor human leukocyte antigen class I (HLA-I) complex	26
12	*esp*	*espfm*, *esp*(fm)	ABQJ01000139.1:70993-76920	Enterococcal surface protein, biofilm formation	5, 23
13	*fms15*		AAAK03000002.1:24432-25460	Adhesin; *E. faecium* surface protein of the MSCRAMM family	6, 21
14	*fnm*		AAAK03000054.1 :14273-15979	Fibronectin binding protein, matrix adhesion	7
15 - 16	General stress protein genes (*gls*)	*gls33* and *gls20* homologous to *gls24* of *E. faecalis*	CP003583.1 :1462262-1462819 CP003583.1 :1467075-1467977	Mutants lacking both *gls33-glsB*, *gls20-glsB1* or both show increased sensitivity for bile salts, maybe important for adaptation to the intestinal environment in addition to virulence. *E. lactis* (clade B) isolates also contain these loci but with lower identity (93-97%)	25
17-18		*glsB* and *glsB1* homologous to *glsB* of *E. faecalis*	AY548799.1 :2876-3118 CP003583.1 :1461988-1462230		

19-21	*lysM*-containing proteins	*lysM1*<br>*lysM2*<br>*lysM3*	CP003351.1:1080180-1080818<br>CP003351.1:509550-510158<br>CP003351.1 :1177130-1177753	Tissue adhesion	18
22		*lysM4*	CP003351.1 :1237718-1238353	Host colonization, role in peptidoglycan synthesis	18
23	*pilA2*	fms21	ABSW01000038.1:25993-27969	Pilus subunit protein A: initial adherence?	10
24	*prpA*		ABQJ01000017.1 :31606-32775	Proline rich protein A, binding to the extracellular matrix proteins fibrinogen and fibronectin	13
25	*ptsD*	*pts*_clin	ABQJ01000092.1 :14293-15114	Phosphotransferase system subunit IID, intestinal colonization determinant during antibiotic treatment	12
26	*sagA*		AF242196.1 :1549-3123	Secreted Antigen A, biofilm formation	14, 15
27	*scm*	*fms10*	CP003583.1:2656348-2658159	Collagen adhesion, MSCRAMM	6
28	*sgrA*	*orf2351*	ABQJ01000055.1:3784-4758	Nidogen-binding surface adhesin implicated in biofilm formation	8
29	*tirE1*		Z_ABQJ01000097.1:c6111-5626	TIR-domain containing protein, promotes survival in blood	16
30	*tirE2*		NZ_ABQJ01000097.1:c8433-7582	TIR-domain containing protein, promotes survival in blood	16

References

1. **Paganelli FL, Willems RJL, Jansen P, Hendrickx A, Zhang X, Bonten MJM, *et al*.** *Enterococcus faecium* Biofilm Formation: Identification of major autolysin $AtlA_{Efm}$, associated Acm surface localization, and $AtlA_{Efm}$-Independent extracellular DNA release. *mBio* 2013;4,e00154-13. DOI: 10.1128/mBio.00154-13
2. **Nallapareddy SR, Singh KV, Murray BE.** Construction of improved temperature-sensitive and mobilizable vectors and their use for constructing mutations in the adhesin-encoding acm gene of poorly transformable clinical *Enterococcus faecium* strains. *Appl Environ Microbiol* 2006;72, 334-345. DOI: 10.1128/AEM.72.1.334-345.2006
3. S**omarajan SR, Roh JH, Singh KV, Weinstock GM, Murray BE.** CcpA is important for growth and virulence of *Enterococcus faecium*. *Infect Immun* 2014;82,3580-3587. DOI: 10.1128/IAI.01911-14
4. **Paganelli FL, Huebner J, Singh KV, Zhang X, van Schaik W, Wobser D, *et al*.** Genome-wide screening identifies phosphotransferase system permease BepA to be involved in *Enterococcus faecium* endocarditis and biofilm formation. *J Infect Dis* 2016;214,189-195. DOI: 10.1093/infdis/jiw108
5. **Willems RJL, Homan W, Top J, van Santen-Verheuvel M, Tribe D, Manzioros X, *et al.*** Variant *esp* gene as a marker of a distinct genetic lineage of vancomycin resistant *Enterococcus faecium* spreading in hospitals. *Lancet* 2001;357,853-855. DOI: 10.1016/S0140-6736(00)04205-7

6. **Sillanpää J, Nallapareddy SR, Prakash VP, Qin X, Höök M, Weinstock GM, *et al.*** Identification and phenotypic characterization of a second collagen adhesin, Scm, and genome-based identification and analysis of 13 other predicted MSCRAMMs, including four distinct pilus loci, in *Enterococcus faecium*. *Microbiol* 2008;154,3199-3211. DOI: 10.1099/mic.0.2008/017319-0
7. **Somarajan SR, La Rose SL, Singh KV, Roh JH, Höök M, Murray BE.** The fibronectin-binding protein Fnm contributes to adherence to extracellular matrix components and virulence of *Enterococcus faecium*. *Infect Immun* 2015;83,4653-4661. DOI: 10.1128/IAI.00885-15
8. **Hendrickx APA, van Luit-Asbrouek M, Schapendonk CME, van Wamel WJB, Braat JC, Wijnands LM, *et al.*** SgrA, a Nidogen-Binding LPXTG Surface Adhesin Implicated in Biofilm Formation, and EcbA, a Collagen Binding MSCRAMM, Are Two Novel Adhesins of Hospital-Acquired *Enterococcus faecium*. *Infect Immun* 2009;77,5097–5106. DOI: 10.1128/IAI.00275-09
9. **Ali L, Spiess M, Wobser D, Rodriguez M, Blum HE, Sakınç T.** Identification and functional characterization of the putative polysaccharide biosynthesis protein (CapD) of *Enterococcus faecium* U0317. *Infect Genet Evol* 2016;37,215-224. DOI: 10.1016/j.meegid.2015.11.020
10. **Hendrickx APA, Bonten MJ, van Luit-Asbrouek M, Schapendonk CME, Kragten AHM, Willems RJL.** Expression of two distinct types of pili by a hospital-acquired *Enterococcus faecium* isolate. *Microbiol* 2008;154,3212-3223. DOI: 10.1099/mic.0.2008/020891-0
11. **Sillanpää J, Nallapareddy SR, Singh KV, Prakash VP, Fothergill T, Ton-That H, *et al.*** Characterization of the *ebp(fm)* pilus-encoding operon of *Enterococcus faecium* and its role in biofilm formation and virulence in a murine model of urinary tract infection. *Virulence* 2010;1,236-246. DOI: 10.4161/viru.1.4.11966
12. **Zhang X, Top J, de Been M, Bierschenk D, Rogers M, Leendertse M, *et al.*** Identification of a genetic determinant in clinical *Enterococcus faecium* strains that contributes to intestinal colonization during antibiotic treatment. *J Infect Dis* 2013;207,1780-1786. DOI: 10.1093/infdis/jit076
13. **Prieto AMG, Urbanus RT, Zhang X, Bierschenk D, Koekman CA, van Luit-Asbrouek M, *et al.*** The N-terminal domain of the thermo-regulated surface protein PrpA of *Enterococcus faecium* binds to fibrinogen, fibronectin and platelets. *Sci Rep* 2016;5,18255. DOI: 10.1038/srep18255
14. **Rangan KJ, Pedicord VA, Wang Y-C, Kim B, Lu Y, Shaham S, *et al.*** A secreted bacterial peptidoglycan hydrolase enhances tolerance to enteric pathogens. *Science* 2016;353,1434-1437. DOI: 10.1126/science.aaf3552
15. **Paganelli FL, de Been M, Braat JC, Hoogenboezem T, Vink C, Bayjanov J, *et al.*** Distinct SagA from hospital-associated clade A1 *Enterococcus faecium* strains contributes to biofilm formation. *Appl Environ Microbiol* 2015;81,6873-6882. DOI: 10.1128/AEM.01716-15
16. **Wagner TM, Janice J, Paganelli FL, Willems RJ, Askarian F, Pedersen T, *et al.*** *Enterococcus faecium* TIR-domain genes are part of a gene cluster which promotes bacterial survival in blood. *Int J Microbiol* 2018;1435820. DOI: 10.1155/2018/1435820
17. **Nallapareddy SR, Weinstock G, Murray BE.** Clinical isolates of *Enterococcus faecium* exhibit strain-specific collagen binding mediated by Acm, a new member of the MSCRAMM family. *Mol Microbiol* 2003;47,1733-1747. DOI: 10.1046/j.1365-2958.2003.03417.x
18. **Cacaci M, Giraud C, Leger L, Torelli R, Martini C, Posteraro B, *et al.*** Expression profiling in a mammalian host reveals the strong induction of genes encoding LysM domain-containing proteins in *Enterococcus faecium*. *Sci Rep* 2018;8,12412. DOI: 10.1038/s41598-018-30882-z
19. **Ali L, Blum HE, Sakınç T.** Detection and characterization of bacterial polysaccharides in drug-resistant enterococci. *Glycoconj J* 2019;36,429-438. DOI: 10.1007/s10719-019-09881-3

20. **Zhang S, Lebreton F, Mansfield MJ, Miyashita S-I, Zhang J, Schwartzman JA, *et al*.** Identification of a botulinum neurotoxin-like toxin in a commensal strain of *Enterococcus faecium*. *Cell Host Microbe* 2018;23,169-176. DOI: 10.1016/j.chom.2017.12.018
21. **Revtovich AV, Tjahjono E, Singh KV, Hanson BM, Murray BE, Kirienko NV.** Development and characterization of high-throughput *Caenorhabditis elegans* - *Enterococcus faecium* infection model. *Front Cell Infect Microbiol* 2021;11:667327. DOI: 10.3389/fcimb.2021.667327
22. **Montealegre MC, Singh KV, Somarajan SR, Yadav P, Chang C, Spencer R, *et al*.** Role of the Emp pilus subunits of *Enterococcus faecium* in biofilm formation, adherence to host extracellular matrix components, and experimental infection. *Infect Immun* 2016;84:1491-1500. DOI: 10.1128/IAI.01396-15
23. **Heikens E, Bonten MJM Willems RJL.** Enterococcal surface protein Esp is important for biofilm formation of *Enterococcus faecium* E1162. *J Bacteriol* 2007;189:8233-8240. DOI: 10.1128/JB.01205-07
25. **Choudhury T, Singh KV, Sillanpää J, Nallapareddy SR, Murray BE.** Importance of two *Enterococcus faecium* loci encoding Gls-like proteins for in vitro bile salts stress response and virulence. *J Infect Dis* 2011;203:1147-1154. DOI: 10.1093/infdis/jiq160
26. **Xiong X, Tian S, Yang P, Lebreton F, Bao H, Sheng K, *et al.*** Emerging *Enterococcus* pore-forming toxins with MHC/HLA-I as receptors. *Cell* 2022;185:1157-71.e22. DOI: 10.1016/j.cell.2022.02.002.

File S3. Prevalence of the Norwegian *E. faecium* cluster types (CT), strain collection, *van*-types, and geographical region.

**CT**	**Number of isolates**	**Strain collection**	***van*-type**	**Geographical region**
ST192-CT3/26	113	VRE 2010-15	*vanB* (*n*=113)	Western
ST117-CT24	51	VRE 2010-15,<br>VSE 2014	*vanB* (*n*=31)	VRE; Western, South-Eastern, Central<br>VSE; All health regions
ST203-CT3061	25	All collections	*vanB* (*n*=3)	VRE; Western, South-Eastern<br>VSE; All health regions
ST80-CT16	23	VRE 2010-15,<br>VSE 2014	*vanB* (*n*=1)	Western, Northern, South-Eastern
ST203-CT20	19	VRE 2010-15	*vanA* (*n*=19)	Central, South-Eastern, Northern
ST203-CT3056	12	VSE 2014		South-Eastern, Western
ST80-CT3097	10	VRE 2010-15	*vanA* (*n*=10)	South-Eastern
ST203-CT3067	9	VSE 2008		South-Eastern, Western, Central
ST203-CT3062	8	VSE 2014, VSE 2008		Central, South-Eastern
ST17-CT3031	8	VSE 2008		South-Eastern
ST202-CT3076	8	VSE 2008		South-Eastern
ST203-CT191	7	VRE 2010-15,<br>VSE 2014	*vanA* (*n*=6)	South-Eastern, Western
ST17-CT3037	7	VRE 2010-15,<br>VSE 2014	*vanA* (*n*=2)	South-Eastern, Northern
ST192-CT3081	6	VSE 2014		South-Eastern
ST117-CT3054	5	VSE 2014		South-Eastern, Northern
ST203-CT3069	4	VSE 2008		South-Eastern, Northern
ST192-CT188	4	VRE 2010-15	*vanA* (*n*=4)	Northern
ST17-CT3030	4	VSE 2008		South-Eastern, Western
ST192-CT1217	4	VSE 2008, VSE 2014		South-Eastern, Central, Northern
ST192_CT397	4	VSE 2008		South-Eastern
ST17-CT1709	2	VRE 1996	*vanB* (*n*=2)	Western
ST192-CT3080	2	VRE 2010-15	*vanB* (*n*=2)	Western, South-Eastern
ST192-CT3082	2	VRE 2010-15	*vanB* (*n*=2)	Western
ST17-CT159	2	VRE 2010-15	*vanB* (*n*=2)	South-Eastern
ST18-CT3042	2	VRE 2010-15	*vanA* (*n*=2)	South-Eastern
Singleton VRE*fm*s and CTs with ≤ 3 isolates	128	All collections	*vanA* (*n*=19)<br>*vanB* (*n*=11)	All health regions

SUPPLEMENTARY RESULTS AND DISCUSSION

Document S1. Details on the major Norwegian VRE*fm* and concurrent major European clusters

ST192-CT3/CT26 *vanB*-type VRE*fm*

The ST192-CT3/CT26 *vanB*-type VRE*fm* (*n*=113) caused the largest outbreak in Norway affecting hospital W1 (*n*=109) and W2 (*n*=4) during 2010-13 (File S1). Before and during the study period 2010-15, *vanB*-type ST192 outbreaks were reported in other countries, including Germany (2008-2009), Denmark (2012-13), and Sweden (2007-2011) [1]. In the global tree, two *vanB*-type VRE*fm* isolates from the Netherlands in 2019 (GCA_900639515.1 (E7654), and GCA_900639525.1 (E7663)) cluster with the Norwegian *vanB*-type ST192 clade (Fig. S7). These two Dutch strains show only fifteen allelic differences compared to the closest Norwegian ST192-CT3. Analyses of German *vanB*-type VRE*fm* sequences retrieved from Sequence Read Archive revealed even closer relatedness to the Norwegian *vanB*-type ST192-CT3/26 with only seven allelic differences between the closest Norwegian isolate from 2011 and the German isolate from 2009 (data not shown). The Norwegian and German isolates also showed more genome similarities between each other compared to the Dutch isolate (Fig. S8).

The integration site of *vanB* carrying Tn*1549* in ST192-CT3/CT26 was identified in an AT rich sequence in the *sir* gene of the *tirE* operon (Table 2) [2]. Tn*1549* insertion in the exact same position in *sir* was also reported in ST192 as well as other STs from Germany [3] indicating this is a preferred integration site. The two Dutch isolates closest to the Norwegian ST192-CT3/CT26 in the global tree, E7654 and E7663 (Fig. S7), have an IS*L3* insertion in the *vanB* cluster and Tn*1549* inserted at the identical AT rich sequence inside the *sir* gene. The high genome sequence identity, a *vanB* gene cluster with identical IS*L3* and insertion site, and the same Tn*1549* insertion sequence support the notion of a common ancestor of *vanB*-type ST192-CT3 from Norway and the Netherlands. The Tn*1549* of the German ST192-CT3 from 2009 shares all the above motioned characteristic with the Norwegian and the Dutch isolates except that the German isolate and one Norwegian isolate from 2011 do not have IS*L3* inserted in Tn*1549* (data not shown). Thus, the German ST192-CT3 may be the common ancestor of *vanB*-type ST192-CT3 from Norway and the Netherlands.

ST117-CT24 *vanB*-type VRE*fm*

In 2013 there was a shift in major cluster in Norway towards the ST117-CT24 *vanB*-type VRE*fm* (*n*=31), also mostly recovered from hospital W1 (*n*=29), belonging to a mixed VRE-VSE cluster encompassing 20 VSE isolates (File S1 and S3). ST117-CT24 *vanB*-type VRE*fm* has been reported in Dutch outbreaks between 2011-17 [4,5], and linked to *vanA*-outbreaks in Denmark and Germany [6,7] as well as linezolid resistance in Austria [8].

The integration site of Tn*1549* in cluster ST117-CT24 occurred in an AT rich sequence in the overlapping 3´end of *btuD* and 5´end of *ndvA* (Table 2) and is thus different from the integration site in ST192-CT3/CT26. Among the closed genomes retrieved from NCBI there were two *vanB*-type VRE*fm* ST117-CT24, one from Norway isolated in 2017 in hospital W1 and one from the Netherlands (GCA_900639505.1 (E7356)) (Fig. S7). The insertion site of Tn*1549* in this Dutch VRE is inside a hypothetical protein gene. Moreover, this Dutch *vanB*-type isolate has IS*30* family transposase (IS*1062*) integrated just upstream of the $vanR_B$ gene, which the Norwegian ST117-CT24 lack. Additionally, other Dutch ST117-CT24 *vanB*-type VRE*fm* outbreak samples from 2014 and 2017 show even more Tn*1549* insertion sites on the chromosome [5]. The overall genetic differences between the ST117-CT24 isolates of Norway and the Netherlands do not support any close genetic relatedness between them.

ST203-CT20 and ST80-CT3097 *vanA*-type VRE*fm*

The ST203-CT20 *vanA*-type VRE*fm* cluster (*n*=19) was recovered from hospitals in Central, South-Eastern, and Northern Norway in 2013-15 while the ST80-CT3097 *vanA*-type VRE*fm* cluster (*n*=10) was found in three hospitals in the South-Eastern Norway in 2010-12 (Fig. S2 and files S1 and S3). A ST80-CT3097 *vanA*-type VRE*fm* cluster has not been reported elsewhere yet. In contrast, ST203-CT20 *vanA*-type VRE*fm* isolates have been reported in Ireland with only seven allelic differences from the Norwegian ST203-CT20 [9]. ST203-CT20 *vanA*-type VRE*fm* has also been reported in blood culture isolates in Germany 2015-18, a vancomycin variable isolate from Sweden [10], and isolates from The Netherlands, Denmark, Belgium, and Australia [9].

References

1. **Pinholt M, Gumpert H, Bayliss S, Nielsen JB, Vorobieva V, Pedersen M, *et al*.** Genomic analysis of 495 vancomycin-resistant *Enterococcus faecium* reveals broad dissemination of a *vanA* plasmid in more than 19 clones from Copenhagen, Denmark. *J Antimicrob Chemother* 2017;72:40-47.
2. **Wagner TM, Janice J, Paganelli FL, Willems RJ, Askarian F, Pedersen T, *et al*.** *Enterococcus faecium* TIR-domain genes are part of a gene cluster which promotes bacterial survival in blood. *Int J Microbiol* 2018;2018:1435820.
3. **Bender J, Kalmbach A, Fleige C, Klare I, Fuchs S, Werner G.** Population structure and acquisition of the *vanB* resistance determinant in German clinical isolates of *Enterococcus faecium* ST192. *Sci Rep* 2016;6:21847.
4. **Zhou X, Chlebowicz MA, Bathoorn E, Rosema S, Couto N, Lokate M, *et al.*** Elucidating vancomycin-resistant *Enterococcus faecium* outbreaks: the role of clonal spread and movement of mobile genetic elements. *J Antimicrob Chemother* 2018;73:3259-3267.
5. **Lisotto P, Couto N, Rosema S, Lokate M, Zhou X, Bathoorn E, *et al*.** Molecular characterisation of vancomycin-resistant *Enterococcus faecium* isolates belonging to the lineage ST117/CT24 causing hospital outbreaks. *Front Microbiol* 2021;12:728356.
6. **Werner G, Neumann B, Weber RE, Kresken M, Wendt C, Bender JK, *et al*.** Thirty years of VRE in Germany – "expect the unexpected": The view from the National Reference Centre for Staphylococci and Enterococci. *Drug Resist Updat* 2020;53:100732
7. **Hammerum AM, Baig S, Kamel Y, Roer L, Pinholt M, Gumpert H, *et al*.** Emergence of *vanA Enterococcus faecium* in Denmark, 2005–15. *J Antimicrob Chemother* 2017;72:2184-2190.
8. **Kerschner H, Cabal A, Hartl R, Machherndl-Spandl S, Allerberger F, Ruppitsch W, *et al*.** Hospital outbreak caused by linezolid resistant *Enterococcus faecium* in Upper Austria. *Antimicrob Resist Infect Control* 2019;8:150.
9. **Egan SA, Kavanagh NL, Shore AC, Mollerup S, Samaniego Castruita JA, O'Connell B, *et al*.** Genomic analysis of 600 vancomycin-resistant *Enterococcus faecium* reveals a high prevalence of ST80 and spread of similar *vanA* regions via IS*1216*E and plasmid transfer in diverse genetic lineages in Ireland. *J Antimicrob Chemother* 2022;77:320-330.
10. **Wagner TM, Janice J, Sivertsen A, Sjögren I, Sundsfjord A, Hegstad K.** Alternative *vanHAX* promoters and increased *vanA*-plasmid copy number resurrect silenced glycopeptide resistance in *Enterococcus faecium. J Antimicrob Chemother* 2021;76:876-882.
